# Multimodal Data for the Detection of Freezing of Gait in Parkinson’s Disease

**DOI:** 10.1038/s41597-022-01713-8

**Published:** 2022-10-07

**Authors:** Wei Zhang, Zhuokun Yang, Hantao Li, Debin Huang, Lipeng Wang, Yanzhao Wei, Lei Zhang, Lin Ma, Huanhuan Feng, Jing Pan, Yuzhu Guo, Piu Chan

**Affiliations:** 1 Xuanwu Hospital of Capital Medical University, Beijing Institute of Geriatrics, Department of Neurology, Neurobiology and Geriatrics, Beijing, 100053 China; 2 The Affiliated Hospital of Xuzhou Medical University, Department of Neurology, Xuzhou, 221006 China; 3 Beihang University, Department of Automation Science and Electrical Engineering, Beijing, 100191 China

**Keywords:** Electroencephalography - EEG, Electromyography - EMG, Parkinson's disease

## Abstract

Freezing of gaits (FOG) is a very disabling symptom of Parkinson’s Disease (PD), affecting about 50% of PD patients and 80% of advanced PD patients. Studies have shown that FOG is related to a complex interplay between motor, cognitive and affective factors. A full characterization of FOG is crucial for FOG detection/prediction and prompt intervention. A protocol has been designed to acquire multimodal physical and physiological information during FOG, including gait acceleration (ACC), electroencephalogram (EEG), electromyogram (EMG), and skin conductance (SC). Two tasks were designed to trigger FOG, including gait initiation failure and FOG during walking. A total number of 12 PD patients completed the experiments and produced a length of 3 hours and 42 minutes of valid data including 2 hours and 14 minutes of normal gait and 1 hour and 28 minutes of freezing of gait. The FOG episodes were labeled by two qualified physicians. The multimodal data have been validated by a FOG detection task.

## Abstract (summary)

**Table:** 

Measurement(s)	electroencephalography (EEG) • electromyography(EMG) • acceleration • skin conductance
Technology Type(s)	electroencephalography (EEG) • electromyography (EMG) • accelerometer • skin conductance

## Background & Summary

Parkinson’s Disease (PD), with more than 10 million patients worldwide, is the second most prevalent brain malady after Alzheimer’s disease^1^. Freezing of gaits (FOG), affecting about 50% of PD patients and 80% of advanced PD patients, as one of the severest manifestations, grievously impacts patients’ life quality and may even menace the lives of aging patients^2^. Therefore, accurate detection or prediction of FOG may significantly improve patients’ life quality and promote personalized treatment of FOG. FOG is an incapacitating issue, which refers to the interruption of the motion caused by the brain’s incompetence to deal with concurrent cognitive and motor request signal input. The complexity of FOG symptoms and its highly-variable manifestations have led to the creation of systems with numerous sensors on various body parts. The physiological data such as Electroencephalogram (EEG), Electrocardiography (ECG), Heart Rate (HR), and Skin Conductance (SC) have been introduced to capture specific features for FOG prediction. A proper application of these data can make an accurate real-time detection or prediction of FOG. Data-driven artificial intelligence methods to accurately detect or even predict FOG have attracted more and more attention. Handojoseno *et al*.^3^ studied the dynamic variation regularity of EEG signal during the occurrence of FOG and implemented detection and prediction of FOG using Bayesian neural networks (BNN). Mazilu *et al*.^4^ utilized SC to discriminate and anticipate FOG. Cole *et al*.^5^ applied dynamic neural network (DNN) to ACC and EMG data to automatically detect FOG. The deep learning (DL) method proposed by JuliÃ Camps *et al*.^6^ provided a new prospective trend of FOG detection: either using traditional machine learning algorithms to extract features and then using DL algorithm to achieve accurate detection of FOG events or directly using end-to-end recurrent neural networks (RNN) to analyze time sequences and achieve FOG detection^7^. All these methods heavily depend on carefully designed experiments and reliable data. Specifically, although DL-based methods provide a promising solution, there are requirements on the amount of data. The existing FOG data sets are limited due to the difficulties and complexities in the simultaneous acquisition of multimodal FOG data. While good results have been reported, automatic and reliable FOG detection and prediction are far from resolved. Due to the limited motion features before FOG occurs, a relatively large and public available multimodal data is desired for the detection and prediction of FOG^8^. A multimodal data for FOG is desired for the detection or prediction of FOG. Acquisition of a sufficient amount of reliable data can be difficult because the simultaneous high-precision acquisition of multiple signals requires complex experimental design and types of equipment. To the best of our knowledge, there is no public-available multimodal database that integrates ACC, EEG, EMG, and SC. In addition, such an intricate system that inevitably reduces the wearability of the data acquisition system can significantly affect patients’ actions and make the experiment deviate from the premise of studying FOG in patients’ daily life settings. It is difficult to solve the dilemma of the stability of data and the portability of sensors during walking tasks. Additionally, as FOG is sporadic, it is the experimental paradigm need to be framed to be able to engender FOGs. Therefore, it is crucial to build a software and hardware platform for multimodal data acquisition in Parkinson’s patients with FOG. Multimodal data provide a comprehensive characterization of the physiological process during the FOG and enable one to reveal the physiological causality of FOG and study personalized interventions. In this study, multimodal data of 12 PD patients with FOG are acquired and analyzed, including the experimental design, sensory system setup, data analysis, and detection of FOG based on multimodal data.

## Methods

The experiments were conducted in Beijing Xuanwu Hospital, China. Ethical approval (No. 2019-014) was obtained from the Ethics Committee of Xuanwu Hospital, Capital Medical University, Beijing, China, and the research was conducted according to the declaration of Helsinki. Written informed consents were obtained from all participants.

### Participants

To conduct the experiments safely and obtain valid data containing sufficient FOG episodes, data were collected in the off-medication state of patients (Off-medication state means the discontinuation of dopamine agonists for over 24 hours and levodopa preparations for over 12 hours). The participants were selected based on the following inclusion and exclusion criteria:

Inclusion criteria:

- Diagnosed as clinically established or probable idiopathic PD according to the Movement Disorder Society Clinical Diagnostic Criteria-2015;
- Experience FOG during the off-time;
- Be able to walk independently during the off-time;

Exclusion criteria:

- No disorders that affect gaits, such as neuropathies, rheumatic and orthopedic disorders;
- No posture problems that affect gaits, such as extreme forward bending or tilting posture;
- No severe vision or hearing loss, dementia (MMSE ≤ 24), or other neurological/orthopedic diseases.

The data have been collected in Beijing Xuanwu Hospital since 2019. Until the paper was written, a total of 18 individuals have been selected based on the inclusion criteria and completed the whole data collection procedures. Among them, data of 12 participants including 6 males and 6 females (13 experiments, Patient ID: 08 conducted the experiment twice on different days) are valid and can be used for the investigation of FOG detection. (The reasons for the “invalid” data include: 1. Patients’ walking posture was affected by wearing the devices due to age reasons or leg disease like gonarthritis; 2. After analyzing the patient’s video, experts concluded that their gait was affected by not only FOG but also other leg diseases; 3. The data were affected by participants’ limited stamina, which leads to poor data quality).

Participants aged between 57 and 81 years (average: 69.1 years), and have disease duration between 1 and 20 years (average: 9.3 years). 10 subjects had conspicuous FOG episodes during the experiments. For detailed information of the 12 participants, please refer to Table 1.

**Table 1:** Participants’ information.

Characteristics		Average (Total)
Age		69.1 ± 7.9
Disease Duration		9.3 ± 6.8
ADL		81.3 ± 16.0
FOG-Q		16.2 ± 4.2
UPDRS	UPDRS-1	10.4 ± 5.5
	UPDRS-2	16.3 ± 10.6
	UPDRS-3	45.0 ± 16.0
	UPDRS-4	2.2 ± 2.9
MMSE		28.2 ± 1.5
MOCA		23.6 ± 3.6
Length of Data(min:sec)		17:05 ± 10:43 (222:03)
Total FOG Time(min:sec)		06:48 ± 7:36 (88:19)
Number of FOG Events		25.7 ± 17 (334)

ADL = Activities of Daily Living Section; UPDRS = Unified Parkinson’s Disease Rating Scales; FOG-Q = Freezing of Gait Questionnaire; MMSE = Mini-Mental State Examination, MOCA = Montreal Cognitive Assessment.

### Data collection

**Fig. 1:**
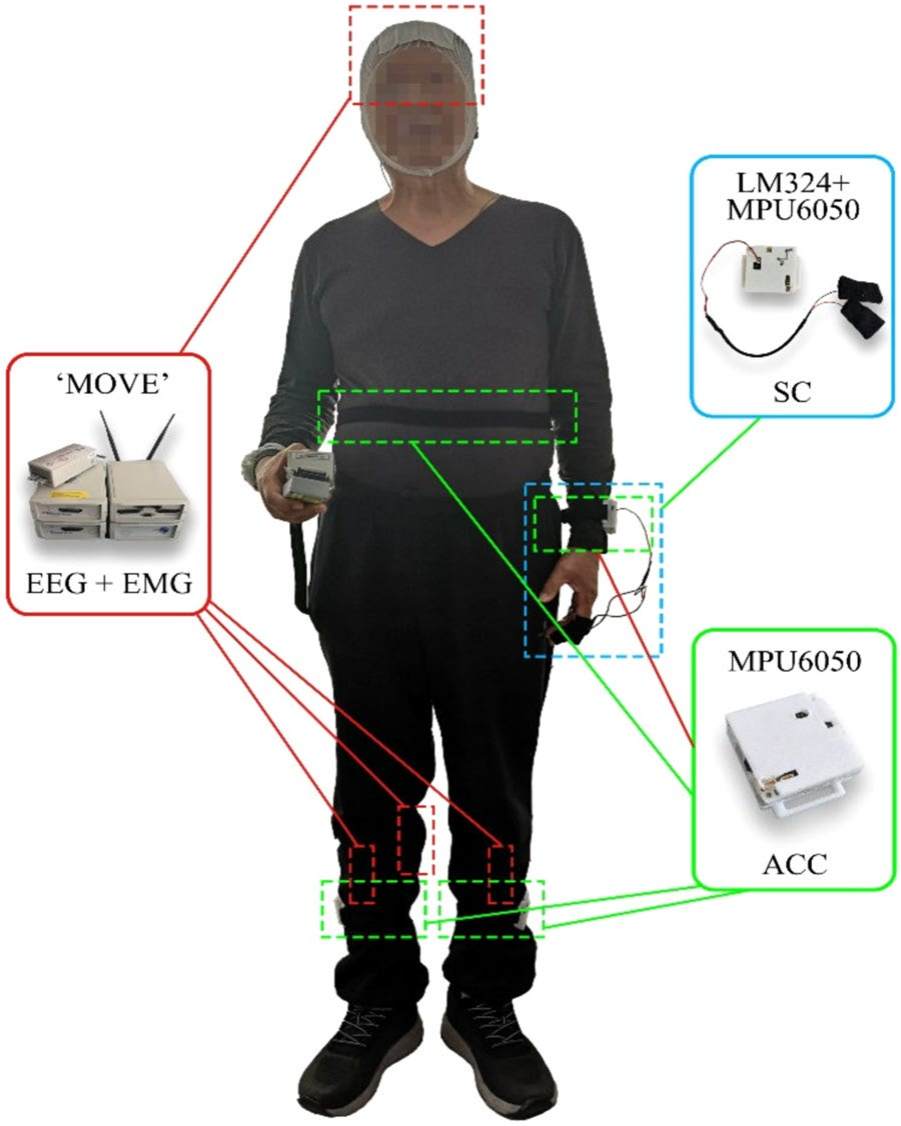
The configuration of the FOG Multimodal data acquisition platform. EEG and EMG were acquired using a 32-channel wireless MOVE system. ACC and SC were acquired using self-designed hardware subsystems based on TDK MPU6050 6-DoF accelerometer and gyro, with STMicroelectronics STM32 processor.

The multimodal sensory platform acquires EEG, EMG, ACC, and SC. The locations of the sensors are shown in Fig. 1.

**Fig. 2:**
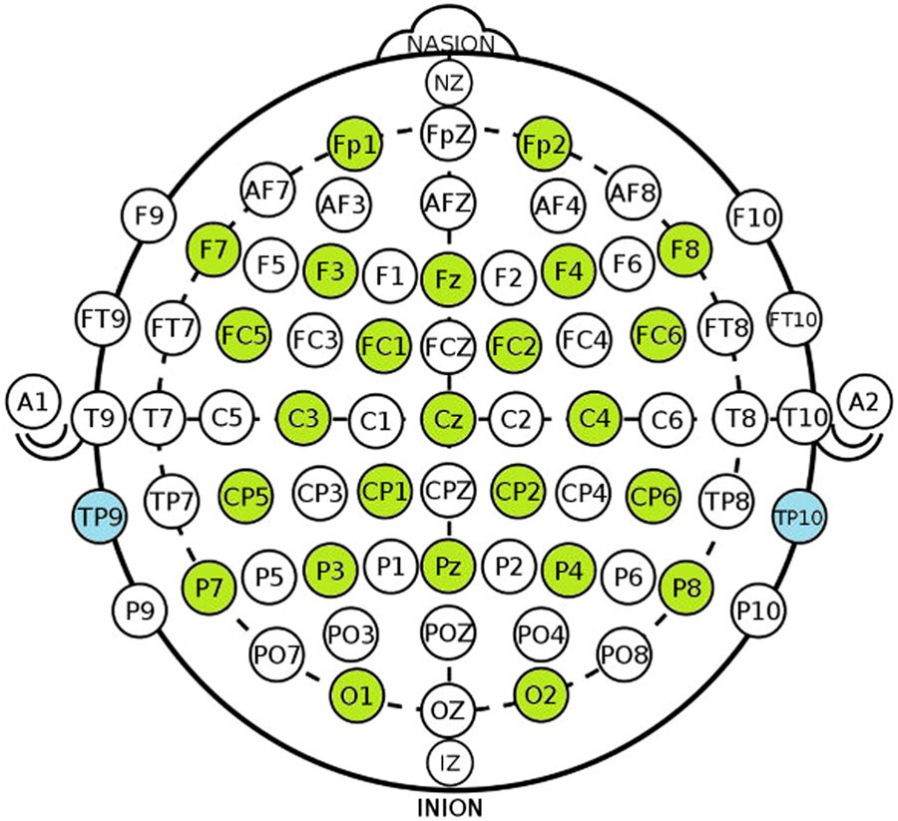
The EEG channels recorded in the international 10–20 system. Channels of 25 EEG signals are painted green. Channels TP9 and TP10 are painted blue, recording the temporal bone’s mastoid process.

**Fig. 3:**
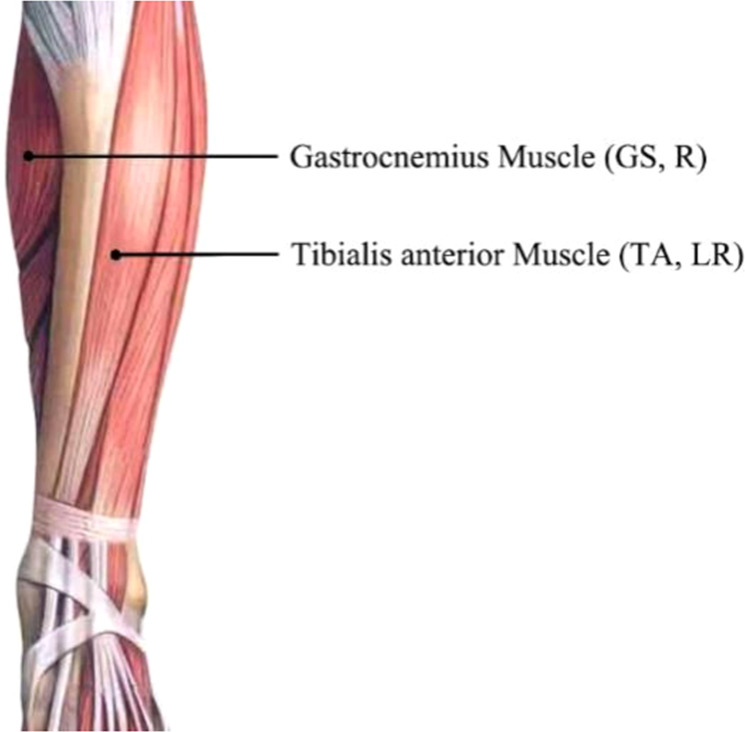
Locations of the EMG sensor. EMG signals were collected at the gastrocnemius (GS) muscle of the right leg and tibia anterior (TA) muscles of both legs.

EEG and EMG were acquired using a 32-channel wireless MOVE system (MOVE, Brain Products GmbH, Gilching, Germany) with a sampling rate of 1000 Hz. Among them, 25 EEG signals were recorded according to the findings that FOG is related to the brain activities in the frontal, parietal, and occipital lobes^9^. Channels TP9 and TP10 recorded the mastoid process of the temporal bone and were used as the reference data preprocessing. Figure 2 shows the definition of the 25 EEG and 2 reference channels in the 10–20 system. The 28^*th*^ Channel was the IO channel which recorded the electrooculogram. The remaining 3 channels of the ‘MOVE’ system were used to collect three channel EMG signals. According to the findings in^10^, the EMG signals were collected at the gastrocnemius (GS) muscle of the right leg and tibia anterior (TA) muscles of both legs, respectively, as shown in Fig. 3.

ACC and SC were acquired using self-designed hardware subsystems based on TDK MPU6050 6-DoF accelerometer and gyro, with STMicroelectronics STM32 processor. Four inertial sensors were mounted at the lateral tibia of the left and right legs, fifth lumbar spine (L5) of the waist and left arm, respectively. The SC acquisition was integrated into the inertial sensor mounted on the left arm. Both SC and ACCs were sampled at 500 Hz and stored on a TF memory card. SC was recorded at the second belly of the left index finger and middle finger. For detailed information, please refer to Table 2.

**Table 2:** Hardware configuration and location of the sensor system.

Sensing Type	System	Sensor Quantity	Sensor Location
28D-EEG	*The wireless* MOVE’	28	37 cm FP1, FP2, F3, F4, C3, C4, P3, P4, O1, O2, F7, F8, P7, P8, Fz, Cz, Pz, FC1, FC2, CP1, CP2, FC5, FC6, CP5, CP6, TP9, TP10, IO
3D-EMG		3	27 cm Gastrocnemius muscle of right leg; Tibialis anterior muscle of left and right legs
3D-accelerometer	MPU6050	4*	27 cm Lateral tibia of left and right legs; Fifth lumbar spine; Wrist
3D-Gyro		4*	
1D-SC	LM324	2	27 cm The second belly of the index finger and middle finger of the left hand

*Not all patients have all 4 inertial sensor data due to the device availability or compliance of patients. Detailed information about each patient is given in Supplemental Table 3 in the supplementary material. TP9, TP10 (signal of the mastoid process of the temporary bone) were used as a reference in data preprocessing. IO (electrooculogram) was given in the dataset without preprocessing.

### Protocol

FOG which can be affected by many factors, such as environments, patients’ emotional states and so on often happen in living circumstance. Tasks that may trigger FOG have been well reported in the literature, including walking through narrow spaces, approaching obstacles, turning, etc.^11^. Based on this knowledge, the experimental procedure was designed to include two tasks to trigger FOGs. During the experiment, the participants overcame FOG by themselves and no intervention has been provided. Data were recorded from the beginning of the experiment through the onset of FOGs, the patient’s overcoming, and the end of the experiment. The procedures of the experiment are summarised as follows:

1. Participants read and sign the informed consent;
2. Participants are asked to take a physical examination and fill in the medical history form and the Unified PD Rating Scale (UPDRS) questionnaires to confirm participants meet the inclusion criteria. Participants do not take any medicine within 2 hours before the experiment to ensure that they were in off-time;
3. Participants wear the multimodal sensory equipment with the help of professional technicians. EEG cap, EMG electrodes, ACC, and SC sensors are mounted at the specified locations as shown in Figs. 1–3 and Table 2;
4. Complete tasks 1–4 defined as follows according to the experimental paradigm. Video is recorded during the whole experiment for physicians to label FOG and non-FOG intervals;
5. Check the saved data at the end of the experiment to confirm no faults in the process. Otherwise, re-do the faulting task after a 2-minute rest.

Two kinds of walking tasks were both completed twice by each participant and are named as task 1–4 respectively. Namely, task 1 is the same as task 2 and task 3 is the same as task 4.

1. Task 1: The walking task was conducted in a setting as shown in Fig. 4. Participants started from a sitting state. When a participant is ready, they rise from a chair at Point A and marches to junction B between the room and a narrow corridor. Turn right and walk into the corridor. Bypass obstacle 1 (can be a chair or a square region on the floor) by turning their bodies. Continue going straight along the narrow corridor until Point C. Make a 180-degree turn at the end of the corridor, and go along an opposite direction. Bypass the three obstacles 1,2 and 3 by turning their body. When they reach the left end of the corridor, Point D, make another 180-degree turn, bypassing obstacles 3 and 2, and reach the door of the room. Enter the room, and walk back to the chair, and sit down. In Fig. 4, the total length of the corridor is about 18 meters and the distance between obstacles 1 and 2, and obstacles 2 and 3 are about 8 meters and 5 meters, respectively. The distance from the chair to the corridor is about 5 meters.

**Fig. 4:**
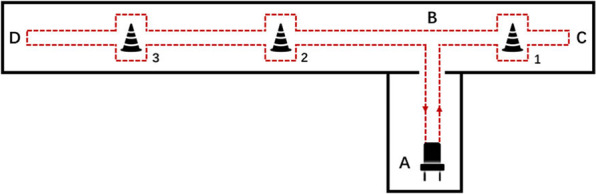
Experimental settings of task 1 and 2. The total length of the corridor is about 18 meters and the distance between obstacles 1 and 2, and obstacles 2 and 3 are about 8 meters and 5 meters, respectively. The distance from the chair to the corridor is about 5 meters.
2. Task 2: Repeat Task 1 one more time.
3. Task 3: This task was conducted in a setting as shown in Fig. 5, where a square was drawn on the ground for patients to make a turn in a limited space. When the patient is ready, stand up from the chair at the end A in the room and march to the pre-pasted square mark at the end B in the room. The participant makes a 180-degree turn in the narrow square region, and then walks straight back to the chair and sits down. In Fig. 5, the length of a side of the square is 0.6 meters and the distance from the chair to the square is 3 meters.

**Fig. 5:**
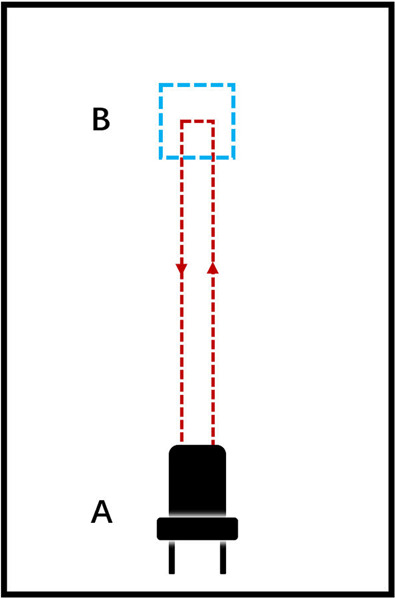
Experimental settings of task 3 and 4. The length of a side of the square is 0.6 meters and the distance from the chair to the square is 3 meters.
4. Task 4: Repeat Task 3. Then end the experiment.

### Data pre-processing

Since the multimodal sensors recorded data separately time alignment of the multimodal data is essential for the consecutive multimodal data analysis and their application in FOG detection. The multimodal data were aligned based on timestamps generated by individual sensory subsystems. Firstly, data of different sampling frequencies will be re-sampled to a unified sampling rate of 500 Hz. That is, the EEG and EMG which were sampled at 1000 Hz were down-sampled to 500 Hz which was the sampling frequency of ACC and SC. A cubic interpolation method was used to calibrate all the singles to the timestamps of ACC subsystems.

The experiments complied with the following rules to simplify the data alignment and annotation:

1. Each acquisition subsystem has its millisecond timer, and the data are parallelly stored using their timestamps. The start and end time of each task were recorded by a separate stopwatch, which was calibrated with the world time, as the world time of each task;
2. All sensors simultaneously sampled data during the whole experiment;
3. The entire process of the experiment was recorded as a video for physicians to label the FOG episodes;
4. All sampling starts at least 30 seconds earlier than each task’s kickoff (the patient was asked to quickly stand up and sit down three times of which the sharp changing ACC was used as the task start instant in the ACC data);
5. Assign the start and end time recorded by the stopwatch to each of the multimodal data and also the video to calibrate the timestamps.

Two qualified physicians from the Department of Neurology, Beijing Xuanwu Hospital labeled the time instances when a FOG started and ended in the video, respectively. The labels were assigned to data points in the aligned multimodal data and completed the Parkinson’s FOG database with expert labels. Artifacts of each mode were removed separately. EEG data were preprocessed using EEGLab and electrooculogram (EOG) artifacts were removed based on the independent component analysis (ICA) with the average of TP9 and TP10 as the reference. The EEG data was then filtered by a band-pass filter of 0.5–100 Hz. EMG data were filtered by a band-pass filter of 10–500 Hz. ACC was filtered by a low-pass filter with a stop frequency of 16 Hz. All the data were filtered out the 50 Hz power line interference by a notch filter and normalized.

## Data Records

### Statistical analysis of FOG

**Fig. 6:**
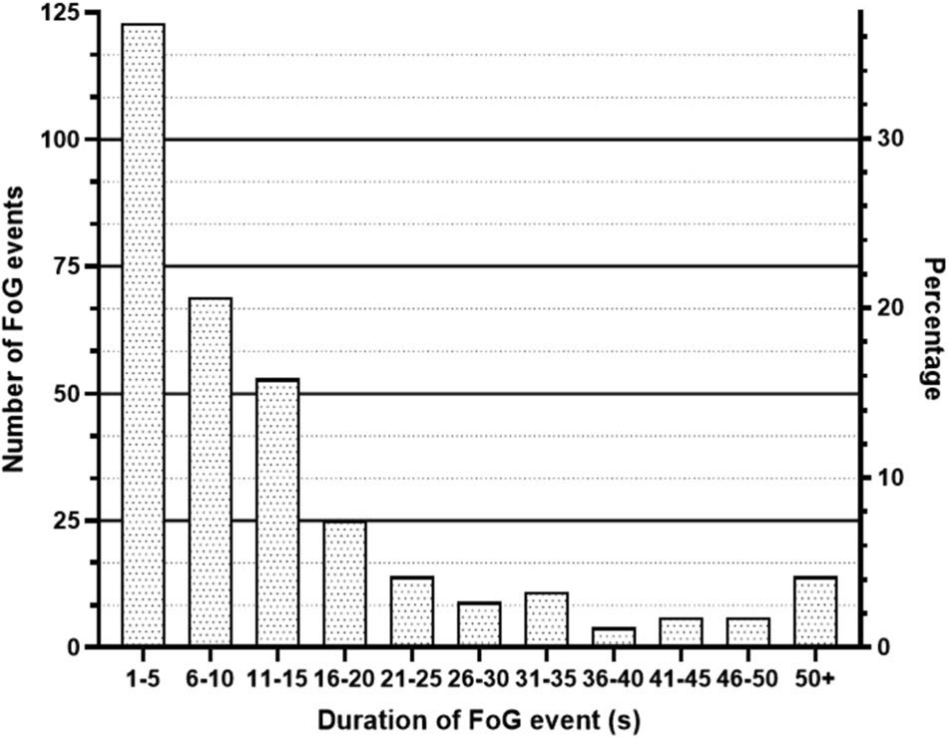
Distribution of FOG Duration. The horizontal axis shows the FOG duration of each event from less than five seconds to more than 50 seconds. The vertical axis shows the number of FOG events and the percentage of the total number of events.

12 PD patients completed 13 valid experiments and produced a length of 3 hours 42 minutes and 3 seconds valid data. There were 334 FOG events with a total FOG duration of 88 minutes and 19 seconds. The duration of each FOG event ranged from 1 to 201 s. Over 35% of episodes lasted less than 5 s, and over 50% of episodes lasted less than 10 s, see Fig. 6 for the distribution of the FOG duration.

The detailed duration of FOG events of each participant is shown in Supplemental Table 1 in the supplementary material. The number of FOG events and the duration of each FOG episode varies significantly among 12 subjects. Patient ID:03 suffered a large amount of FOG during data collection while Patient (ID:02) showed a few; most FOG events of Patient ID:09 have a duration less than 5 seconds, while Patient ID:10 have many FOG events that last more than 40 seconds. Such noticeable inter-subject variability in FOG events indicates that results of the subject-specific study of FOG detection and prediction can be better than those of subject-independent study.

Both the raw data directly obtained from the hardware system, and the filtered data which have been preprocessed and labeled, are published on Mendeley Data. The specific description of the dataset can be referred to supplementary materials or online dataset description files. Raw data available at^12^, filtered data available at^13^.

### Data description

Combined with the standardized experimental paradigm we designed, which can effectively induce FOG, each patient was asked to finish four tasks, including quarter turns, 180-degree turn, and bypass obstacles. EEG, EMG, ECG, skin conductance and acceleration data were collected during the task. With the video recording, two sophisticated doctors would label the data to indicate whether there have FOG occurred. It is divided into raw data and filtered data. There are 12 sub-folders in each folder, representing the data of 12 different patients. ID:008 has done two experiments and is divided into 1/2 in its sub-folder. The two experiments were not completed on the same day. The two experiments were well separated but with exactly the same settings so that the participants were not tired and the results would not be affected by fatigue.

Raw data were collected in each experiment, divided into data collected by the ‘MOVE’ system, including EEG, EMG and ECG with the sampling frequency of 1000 Hz, and the SC and ACC data collected by MPU6050 and LM324 with the sampling frequency of 100 Hz. Data collected by the ‘MOVE’ system is saved using the suffix ‘.eeg’, ‘.vhdr’, and ‘.vmrk’, while the filename is random. These three files are generated directly by the ‘MOVE’ system. EEGLab can read them. Raw SC and ACC data are saved using the suffix ‘.csv’. And the filename is the location of the sensor. For example, ‘LShank.csv’ contains the raw acceleration data collected from the left shank. For eight columns in each CSV file, the order of data is ‘timestamps, accelerometer-x, accelerometer-y, accelerometer-z, Gyro-x, Gyro-y, Gyro-z, NC/SC’. The eighth column of the ‘LShank.csv’, ‘RShank.csv’, and ‘Waist.csv’ is NC, which is invalid data. The eighth column of ‘Arm.csv’ is skin conductance.

Filtered data has been labeled, sliced, and preprocessed. The *n*^th^ task’s data file of each experiment is named ‘task_n.txt’. In the txt file array, the vertical axis represents the time with the sampling frequency of 500 Hz. There are 60 columns on the horizontal axis. The first column is Time-stamps, which begin from 0. Columns 2 to 26 are EEG signals, including FP1, FP2, F3, F4, C3, C4, P3, P4, O1, O2, F7, F8, P7, P8, Fz, Cz, Pz, FC1, FC2, CP1, CP2, FC5, FC6, CP5, CP6. Columns 27 to 31 are EMG, ECG, and Electrooculogram signals, the order of each subject is given in Supplemental Table 2 in the supplementary material. Columns 32 to 59 are 28 columns (7 by 4) of acceleration data on the left shank, right shank, waist, and arm. For every 7 columns, the order of data is ‘accelerometer-x, accelerometer-y, accelerometer-z, Gyro-x, Gyro-y, Gyro-z, NC/SC’. The seventh column of the first three is NC, which is invalid data. The seventh column of the fourth is SC (SC is collected by the sensor on the arm only). Please notice that some patients do not have all 4 inertial sensor data (refer to the Supplemental Table 3 for details). Those not available inertial information are denoted as zeros in the data file. The last column is Label. Label 1 indicates the presence of FOG, and 0 indicates the FOG-free.

## Technical Validation

Literature has shown that multimodal information benefits the accurate detection of FOG. In this section, the collected multimodal data were used to compare the performance of each unimodal data in the task of FOG detection. Data from different sources were time-aligned and preprocessed to remove the effect of artifacts. Features were extracted for each single modal data and Support vector machine (SVM) classifiers with radial basis function kernels were trained based on unimodal sensing data and their combinations.

### Data segmentation and assignment of labels

In the FOG detection study, the multimodal FOG data were segmented according to the expert labels and assigned a common label for each segment. The data were segmented using a sliding window method with a window length of 3 seconds and a sliding step size of 0.3 seconds. Each segment was assigned a common label based on the proportion of FOG time points, that is, the Percentage of FOG (PFG) points defined as (1).

1 $$PFG=\frac{{N}_{FOG}}{{N}_{FOG}+{N}_{N}}\ast 100{\rm{ \% }}$$

where *N*_*FOG*_ is the number of FOG data points in the segment, while *N*_*N*_ is the number of FOG-free points. The label of the segment is determined by (2), where T is the appropriate threshold selected by the researcher, which is usually around 0.75–0.85.

2 $$SegmentLabel=\left\{\begin{array}{ll}1, & FPG\ge T\\ -1, & FPG < T\end{array}\right.$$

The labeling threshold was set to 80% in the following discussion, which means that FOG’s appearance in the data segment if over 80% data points were labeled as positive by physicians. Each segment of the multimodal data and the associated segment label is composed of an effective sample for the following features extraction and classification.

### Feature extraction

A total number of sixteen statistical features (as in Table 3) were employed for the classification of FOG according to the results in references. Each unimodal data was used to detect FOG individually and the multimodal data were then used by combining all these features in the detection of FOG.

**Table 3:** Multimodal features and brief description.

Data	Channel Quantity	Feature	Description
EEG	25	*WE*_*δ*_	Represents changes in energy of FOG and locomotion period of PD patients’ EEG signal
		*WE*_*θ*_	
		*WE*_*α*_	
		TWE	Represents changes in energy complexity
EMG	3	MAV	Estimation of the STD of EMG signals
		ZC	Related to the frequency of EMG signals
		SSC	
		WL	Directly related to the EMG signals
ACC	3	SE	Evaluate the repeatability of the waveform
		STD	Standard Deviation
		TP	Detection algorithm of FOG proposed by Moore *et al*.^15^
		FI	
SC	3*	MAV	Evaluate the mean value of the waveform
		STD	Standard Deviation
		MED	Related to the amplitude and frequency of SC signals
		MIN	
		MAX	
		ZC	

The definition of the abbreviations are TWE - total wavelet entropy, MAV - mean absolute value, ZC - zeros crossing, SSC - slope sign change, WL - wave length, SE - sample entropy, STD - standard deviation, TP - total power, FI - freezing index, MED - median value, MIN - minimum value, MAX - maximum value. *The other two channels of SC signals are obtained by taking the first-order and second-order derivatives of the first channel.

For the EEG mode, 5-scale discrete wavelet transforms (DWT) were applied to each of the 25 channels to obtain five rhythms, that is, the *δ* wave (0–3.9 Hz); *θ* wave (3.9–7.8 Hz); *α* wave (7.8–15.6 Hz); *β* wave (15.6–31.3 Hz); and *γ* wave (31.3–62.5 Hz)^14^. The extracted EEG features of each channel were wavelet energy (WE) of a segement of data in *δ*, *θ*, *α*-bands and the associated total wavelet entropy (TWE), denoted as *WE*_*δ*_, *WE*_*θ*_, *WE*_*α*_, and TWE, respectively. The WE of each component are defined as (3)

3 $$W{E}_{j}=\mathop{\sum }\limits_{k=1}^{N}| {y}_{j}{| }^{2}$$

Where *y*_*j*_ denotes the *j*_*th*_ components of an EEG channel; *WE*_*j*_ denotes the WE of the *j*_*th*_ component of an EEG channel after the DWT; N is the window length of a segment. The associated TWE is defined as (4)

4 $$TWE=-\sum _{j}\frac{W{E}_{j}}{{\sum }_{j}W{E}_{j}}{\rm{\log }}\frac{W{E}_{j}}{{\sum }_{j}W{E}_{j}}$$

For the EMG data, four features were extracted for each channel of EMG, including Mean Absolute Value (MAV), Zeros Crossing (ZC), Slope Sign Change (SSC), and Wave Length (WL).

For the ACC data, four features were extracted from the three direction accelerations which were measured at lateral tibia of the left or right legs as the associated Sample Entropy (SE), Standard Deviation (STD), Total Power (TP), and Freezing Index (FI) which is defined as the ratio of the powers in freezing band (3–8 Hz) and in locomotion band (0.5–3 Hz)^15^.

For the SC data, the multiplicative inverse was applied to the original signal and produced the first time series, then the first- and second-order derivatives were defined as the second and third time series. Six features were extracted from each time series as Mean Absolute Value (MAV), Standard Deviation (STD), Median Value (MED), Minimum Value (MIN), Maximum Value (MAX), and Zeros Crossing (ZC).

### FOG detection

A total number of 15 combinations were considered by exploring all combinations of EEG, EMG, ACC, and SC features. The dataset was divided into training and test sets with a quantity ratio of 4:1 randomly. A grid search method was used to determine the hyper-parameters of the SVM model based on cross-validation performance. Replicating each experiment 10 times, the average values of the performance, including accuracy, sensitivity, specificity, precision, F1 value, area under curve (AUC) were reported to evaluate the classification performance. The performance of the different modal data was compared under subject-dependent setting, namely, the performance of the multimodal data was evaluated individually for each subject.

**Fig. 7:**
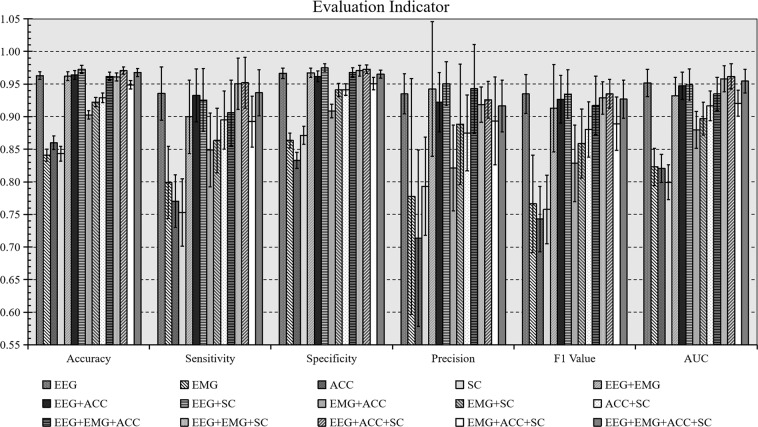
Average Result of four types of SVM classification in subject-dependent analysis. A total number of 15 combinations were considered by exploring all combinations of EEG, EMG, ACC, and SC features. The average values of the performance, including accuracy, sensitivity, specificity, precision, F1 value, and area under curve (AUC) were reported to evaluate the classification performance.

The performance of each combination is shown in Fig. 7. The results of FOG detection showed that the average values of all criteria in all mode combinations exceeded 90%. The average accuracies and AUCs of the 15 modal combinations exceeded 93% and 90%, respectively. The EEG data performed the best in four unimodal data and EMG performed the worst classification results. The multimodal data which combined more than one mode significantly surpassed the performance of single-mode features. This indicates that multimodal data characterized FOG better than single modal data did. The multimodal data performed better when EEG was included, in which the combination of EEG, ACC, and SC performed the best FOG detection.

Detailed results of the subject-specific analysis can be obtained from Supplemental Table 4 in the supplementary material.

## Usage Notes

Due to the different duration of FOG events, there will be a class imbalance when the data are used in a machine learning task. Therefore, class rebalancing adjustment is recommended. Although we address the impact of class imbalance by adjusting the class weights of the classifiers, it can be observed that the duration of FOG events in the data still had a significant impact on the classification performance, especially for the patient either with a majority of FOG (such as subject ID:03) or minority of FOG events (such as subject ID:09). A data rebalancing operation is strongly recommended for subject ID:09, which has the most severe data imbalance. Subject ID:02 and subject ID:05 barely showed any FOG in the experiment. Although these data can still provide useful information, users can discard these two sets of data in the analysis.

The comprehensive comparison showed that the EEG signals have the best performance in the detection of FOG than ACC, EMG, and SC did. However, the preparation, acquisition, and preprocessing of EEG data can be costly and time-consuming. Therefore, EEG does not suit for long-term monitoring of FOG in a living condition even though it produced the best performance. It is worthy to explore the dynamic dependence among the multimodal data and develop an easy-to-implement long-term FOG monitoring method. This will be worthy to further study.

## Supplementary information

Supplemental Table 2

Supplemental Table 4

UPDRS, FOG-Q, MMSE, and MOCA

DATA QUALITY CHECK REPORT

Supplemental Table 1

Supplemental Table 3

## Data Availability

The preprocessing of EEG data was conducted with EEGLab. The codes of the preprocessing of EMG and ACC, labeling of raw data, and feature extraction are available with the data file^13^.
