# Supplementary material for: Multimodal Data for the Detection of Freezing of Gait in Parkinson’s Disease
Source: Sci Data. 2022 Oct 7;9:606. doi: 10.1038/s41597-022-01713-8 (PMC9546845; doi:10.1038/s41597-022-01713-8)
Supplement: Supplementary file 1 — Supplemental Table 2 [file 41597_2022_1713_MOESM1_ESM.pdf]

Supplemental Table 2. Order of 27-31 Columns

Patient ID	27(EMG-1)	28(EMG-2)	29	30(EMG-3)	31(EMG-4)
1	R-TA	L-TA		ECG	R-GS
2	R-TA	L-TA		ECG	R-GS
3	L-TA	R-TA		ECG	R-GS
4	L-TA	R-TA		ECG	R-GS
5	L-TA	R-TA		ECG	R-GS
6	R-TA	L-TA		ECG	R-GS
7	R-TA	L-TA	IO	ECG	R-GS
008-1	R-TA	L-TA		ECG	R-GS
008-2	R-TA	R-GS		ECG	L-TA
9	L-TA	R-TA		R-GS	ECG
10	L-TA	R-TA		ECG	R-GS
11	L-TA	R-TA		ECG	R-GS
12	L-TA	R-TA		ECG	R-GS

* R-TA => EMG of Tibialis anterior muscle of right leg
R-GS => EMG of Gastrocnemius muscle of right leg
L-TA => EMG of Tibialis anterior muscle of left leg
IO => Electrooculogram
