# Supplementary material for: Multimodal Data for the Detection of Freezing of Gait in Parkinson’s Disease
Source: Sci Data. 2022 Oct 7;9:606. doi: 10.1038/s41597-022-01713-8 (PMC9546845; doi:10.1038/s41597-022-01713-8)
Supplement: Supplementary file 2 — Supplemental Table 4 [file 41597_2022_1713_MOESM2_ESM.pdf]

Supplemental Table 4. Results of subject-specific analysis with different feature combinations							
Patient ID	Features	Accuracy	Sensitivity	Specificity	Precision	F1 Value	AUC
	EEG	0.9624±0.0056	0.9272±0.0168	0.9787±0.003	0.9534±0.0048	0.9398±0.0093	0.953±0.0085
	EMG	0.8822±0.006	0.9017±0.0159	0.8731±0.0063	0.7687±0.0095	0.8295±0.0092	0.8874±0.008
	ACC	0.8821±0.012	0.925±0.0352	0.8621±0.0102	0.7585±0.0131	0.833±0.0183	0.8936±0.0174
	SC	0.8092±0.018	0.7094±0.0414	0.8557±0.0145	0.6967±0.0271	0.7023±0.0311	0.7825±0.0234
	EEG+EMG	0.9528±0.0089	0.904±0.016	0.9755±0.008	0.9455±0.0169	0.9239±0.0144	0.9397±0.0104
	EEG+ACC	0.9553±0.0071	0.921±0.0128	0.9713±0.006	0.9377±0.0129	0.9291±0.0113	0.9462±0.0084
	EEG+SC	0.9615±0.0047	0.9283±0.0128	0.977±0.003	0.9499±0.0071	0.9387±0.0079	0.9527±0.0067
1	EMG+ACC	0.9254±0.007	0.9283±0.0103	0.924±0.008	0.8512±0.0141	0.8877±0.0104	0.9262±0.0073
	EMG+SC	0.9279±0.0067	0.9144±0.0172	0.9341±0.0064	0.8667±0.0114	0.8896±0.0104	0.9243±0.0089
	ACC+SC	0.9249±0.0094	0.91±0.0149	0.9318±0.0083	0.862±0.0162	0.885±0.0146	0.9209±0.0106
	EEG+EMG+ACC	0.9554±0.0056	0.9212±0.0078	0.9713±0.0062	0.9379±0.0124	0.9291±0.0087	0.9462±0.0058
	EEG+EMG+SC	0.9584±0.007	0.9352±0.0166	0.9691±0.0038	0.9343±0.0088	0.9345±0.0116	0.9521±0.0095
	EEG+ACC+SC	0.9569±0.0061	0.9328±0.0128	0.9681±0.0045	0.9319±0.0105	0.9321±0.0099	0.9504±0.0077
	EMG+ACC+SC	0.9465±0.0059	0.9116±0.0143	0.9628±0.0055	0.9199±0.0109	0.9154±0.0096	0.9372±0.0076
	EEG+EMG+ACC+SC	0.9569±0.006	0.9126±0.0134	0.9775±0.004	0.9502±0.0081	0.9307±0.0099	0.9451±0.0078
2*	/	/	/	/	/	/	/
	EEG	0.9735±0.0039	0.9821±0.0027	0.9341±0.0172	0.9856±0.0037	0.9839±0.0023	0.9581±0.0088
	EMG	0.8604±0.0063	0.9336±0.0049	0.5245±0.0252	0.9002±0.0048	0.9166±0.0037	0.729±0.013
	ACC	0.8772±0.0056	0.8998±0.0052	0.7735±0.0224	0.9481±0.0048	0.9233±0.0035	0.8366±0.0113
	SC	0.9087±0.0087	0.9556±0.0077	0.6933±0.0197	0.9348±0.0042	0.945±0.0054	0.8245±0.0123
	EEG+EMG	0.9684±0.0039	0.9743±0.0037	0.9412±0.0087	0.9871±0.0019	0.9806±0.0024	0.9578±0.0053
	EEG+ACC	0.9713±0.0047	0.9842±0.0037	0.9119±0.014	0.9809±0.003	0.9826±0.0028	0.9481±0.008
	EEG+SC	0.9821±0.0028	0.9868±0.0029	0.9605±0.0095	0.9914±0.0021	0.9891±0.0017	0.9736±0.0049
3	EMG+ACC	0.9088±0.0057	0.9331±0.0059	0.7973±0.0216	0.9549±0.0045	0.9438±0.0036	0.8652±0.0108
	EMG+SC	0.9485±0.0061	0.9728±0.0044	0.8365±0.0231	0.9647±0.0049	0.9688±0.0037	0.9047±0.0122
	ACC+SC	0.9505±0.0056	0.9599±0.0061	0.9069±0.0092	0.9794±0.002	0.9695±0.0035	0.9334±0.006
	EEG+EMG+ACC	0.9687±0.0029	0.9805±0.0025	0.9144±0.014	0.9814±0.003	0.9809±0.0017	0.9474±0.0068
	EEG+EMG+SC	0.974±0.0034	0.9908±0.0026	0.8971±0.02	0.9779±0.0042	0.9843±0.002	0.9439±0.0096
	EEG+ACC+SC	0.9791±0.0036	0.9888±0.0036	0.9343±0.0145	0.9858±0.0031	0.9873±0.0022	0.9616±0.0072
	EMG+ACC+SC	0.9585±0.0052	0.9686±0.0045	0.9122±0.0205	0.9807±0.0044	0.9746±0.0032	0.9404±0.0105
	EEG+EMG+ACC+SC	0.9637±0.0049	0.9602±0.0052	0.9794±0.0091	0.9954±0.002	0.9775±0.0031	0.9698±0.0058
	EEG	0.9764±0.0043	0.9658±0.0179	0.9786±0.0048	0.9061±0.0195	0.9346±0.0117	0.9722±0.0086
	EMG	0.9155±0.008	0.7452±0.0344	0.9515±0.0066	0.7663±0.0262	0.7544±0.0253	0.8484±0.0174
	ACC	0.8767±0.0121	0.8038±0.0472	0.8921±0.0078	0.6135±0.0251	0.6951±0.0323	0.848±0.0252
	SC	0.9215±0.014	0.6605±0.0613	0.9767±0.006	0.8581±0.0398	0.7449±0.0518	0.8186±0.0323
	EEG+EMG	0.9782±0.0027	0.9269±0.0226	0.9891±0.0029	0.9478±0.0125	0.9368±0.0085	0.958±0.0103
	EEG+ACC	0.9766±0.0052	0.9462±0.0238	0.983±0.0027	0.9224±0.0123	0.9337±0.0152	0.9646±0.0123
	EEG+SC	0.9812±0.0091	0.9468±0.0445	0.9884±0.0033	0.9463±0.0159	0.9459±0.0273	0.9676±0.0229
4	EMG+ACC	0.9386±0.0108	0.8332±0.0403	0.9609±0.0086	0.82±0.0337	0.8257±0.0309	0.8971±0.0213
	EMG+SC	0.9581±0.0072	0.9247±0.0259	0.9652±0.007	0.8502±0.0263	0.8852±0.0198	0.945±0.0133
	ACC+SC	0.9564±0.0083	0.9256±0.0288	0.9629±0.0079	0.8417±0.0277	0.8811±0.0219	0.9443±0.015
	EEG+EMG+ACC	0.9762±0.0054	0.9032±0.0239	0.9917±0.0033	0.9588±0.016	0.9298±0.0165	0.9474±0.0123
	EEG+EMG+SC	0.9752±0.0074	0.9715±0.0185	0.976±0.0065	0.8964±0.0263	0.9322±0.0198	0.9738±0.011
	EEG+ACC+SC	0.9785±0.0072	0.9266±0.0352	0.9894±0.0035	0.9495±0.0166	0.9373±0.0219	0.958±0.018
	EMG+ACC+SC	0.9665±0.0075	0.9212±0.0315	0.976±0.0068	0.8915±0.028	0.9056±0.0216	0.9486±0.0158
	EEG+EMG+ACC+SC	0.9761±0.007	0.9636±0.0196	0.9788±0.0067	0.9066±0.0271	0.9339±0.0188	0.9712±0.0109
5*	/	/	/	/	/	/	/
	EEG	0.9552±0.0084	0.8944±0.0318	0.9785±0.0049	0.9412±0.0117	0.9169±0.0169	0.9364±0.0153
	EMG	0.891±0.0077	0.8735±0.0229	0.8978±0.0052	0.7666±0.0108	0.8162±0.0142	0.8856±0.012
	ACC	0.7945±0.0085	0.603±0.0272	0.8679±0.0066	0.6367±0.0137	0.6187±0.0198	0.7354±0.0137
	SC	0.8016±0.0064	0.7115±0.0198	0.8361±0.0098	0.6255±0.0116	0.6651±0.0111	0.7738±0.0086
	EEG+EMG	0.9687±0.0057	0.9558±0.0141	0.9737±0.0064	0.9335±0.015	0.9443±0.0102	0.9647±0.0074
	EEG+ACC	0.9531±0.0073	0.9293±0.0165	0.9622±0.0098	0.9048±0.0218	0.9166±0.0123	0.9458±0.0084
	EEG+SC	0.9624±0.0057	0.9402±0.0152	0.9709±0.0058	0.9258±0.0137	0.9326±0.0105	0.9555±0.0079
6	EMG+ACC	0.9115±0.0066	0.9064±0.0188	0.9134±0.0111	0.8012±0.0186	0.8501±0.0103	0.9099±0.0077
	EMG+SC	0.9297±0.0056	0.8947±0.0216	0.9432±0.0048	0.8583±0.0102	0.8758±0.0109	0.9189±0.0101
	ACC+SC	0.9082±0.0051	0.814±0.0185	0.9443±0.0052	0.849±0.0108	0.8307±0.0104	0.8791±0.0086
	EEG+EMG+ACC	0.9622±0.0055	0.9217±0.0126	0.9778±0.0053	0.9413±0.0136	0.9311±0.01	0.9497±0.0072
	EEG+EMG+SC	0.9673±0.0072	0.9334±0.0241	0.9803±0.0043	0.9481±0.0108	0.9405±0.0137	0.9569±0.0121
	EEG+ACC+SC	0.9648±0.005	0.9283±0.018	0.9788±0.0038	0.9441±0.0092	0.9358±0.0098	0.9535±0.0087
	EMG+ACC+SC	0.9381±0.0069	0.8912±0.0197	0.9561±0.0072	0.8867±0.0162	0.8886±0.0129	0.9237±0.0099
	EEG+EMG+ACC+SC	0.9577±0.0095	0.8924±0.0288	0.9827±0.0041	0.9523±0.0124	0.921±0.0188	0.9376±0.0153
	EEG	0.9491±0.0094	0.9225±0.0155	0.9653±0.0095	0.9422±0.0159	0.932±0.0129	0.9439±0.0102
	EMG	0.8389±0.0188	0.7829±0.0386	0.873±0.0137	0.7912±0.0219	0.7865±0.0276	0.828±0.0221
	ACC	0.8548±0.0161	0.8346±0.017	0.8672±0.0179	0.7941±0.0244	0.8135±0.0198	0.8509±0.0159
	SC	0.8924±0.0104	0.8665±0.0254	0.9083±0.0177	0.8534±0.0221	0.8594±0.0136	0.8874±0.0114
	EEG+EMG	0.9449±0.0097	0.9145±0.022	0.9634±0.0087	0.9389±0.014	0.9263±0.0133	0.939±0.0117

7	EEG+ACC	0.9608±0.0107	0.959±0.0214	0.9619±0.013	0.9396±0.0189	0.9489±0.0141	0.9605±0.0118
	EEG+SC	0.966±0.0116	0.965±0.0161	0.9666±0.0113	0.9467±0.0174	0.9556±0.0151	0.9658±0.0122
	EMG+ACC	0.9013±0.0063	0.8958±0.0168	0.9047±0.0118	0.8525±0.0136	0.8732±0.0079	0.9002±0.0069
	EMG+SC	0.9236±0.0086	0.8884±0.0151	0.9452±0.0139	0.9088±0.0207	0.8981±0.0109	0.9168±0.0084
	ACC+SC	0.9406±0.0071	0.9471±0.0119	0.9366±0.0114	0.9018±0.0153	0.9237±0.0087	0.9418±0.0068
	EEG+EMG+ACC	0.9551±0.0109	0.9336±0.0199	0.9682±0.0085	0.9477±0.0139	0.9403±0.0146	0.9509±0.0124
	EEG+EMG+SC	0.9616±0.0086	0.957±0.0127	0.9644±0.0094	0.9429±0.0145	0.9497±0.0111	0.9607±0.009
	EEG+ACC+SC	0.9668±0.0063	0.9713±0.013	0.9641±0.0073	0.9433±0.0104	0.957±0.0082	0.9677±0.0072
	EMG+ACC+SC	0.9452±0.0079	0.9432±0.0111	0.9464±0.0121	0.9154±0.0171	0.9289±0.0097	0.9448±0.0074
	EEG+EMG+ACC+SC	0.9598±0.0068	0.9585±0.0096	0.9606±0.0087	0.9374±0.013	0.9476±0.0087	0.9595±0.0068
8(1)	EEG	0.9655±0.0047	0.9525±0.007	0.9759±0.0074	0.9693±0.0088	0.9608±0.0053	0.9642±0.0046
	EMG	0.8895±0.0077	0.8636±0.0129	0.9101±0.0101	0.8847±0.0114	0.8738±0.0089	0.8868±0.0079
	ACC	0.8993±0.0072	0.8986±0.0131	0.8998±0.0066	0.8775±0.0072	0.8877±0.0084	0.8992±0.0076
	SC	0.7712±0.0144	0.7481±0.0132	0.7895±0.0223	0.7393±0.0215	0.7434±0.0143	0.7688±0.0137
	EEG+EMG	0.9635±0.0051	0.9483±0.0094	0.9755±0.008	0.9688±0.0097	0.9583±0.0057	0.9619±0.0051
	EEG+ACC	0.9683±0.006	0.9647±0.0093	0.9711±0.0083	0.964±0.01	0.9643±0.0068	0.9679±0.006
	EEG+SC	0.9734±0.0044	0.9663±0.0077	0.979±0.0056	0.9736±0.0069	0.9698±0.005	0.9727±0.0045
	EMG+ACC	0.9342±0.0054	0.9247±0.0093	0.9417±0.0076	0.9271±0.0094	0.9256±0.006	0.9332±0.0055
	EMG+SC	0.9409±0.0053	0.9266±0.0139	0.9523±0.0104	0.9395±0.0119	0.9328±0.0063	0.9394±0.0056
	ACC+SC	0.9395±0.0078	0.9417±0.015	0.9378±0.0119	0.9238±0.0133	0.9324±0.0087	0.9398±0.008
	EEG+EMG+ACC	0.9686±0.0065	0.9583±0.0111	0.9768±0.0066	0.9706±0.0081	0.9644±0.0074	0.9676±0.0068
	EEG+EMG+SC	0.9719±0.0049	0.96±0.0099	0.9815±0.0053	0.9764±0.0066	0.9681±0.0057	0.9707±0.0053
	EEG+ACC+SC	0.9734±0.0045	0.9621±0.008	0.9824±0.0035	0.9776±0.0044	0.9697±0.0052	0.9723±0.0048
	EMG+ACC+SC	0.9585±0.0056	0.9587±0.0084	0.9584±0.0084	0.9485±0.0098	0.9535±0.0062	0.9586±0.0055
	EEG+EMG+ACC+SC	0.9708±0.0044	0.9564±0.006	0.9822±0.0044	0.9772±0.0055	0.9667±0.005	0.9693±0.0045
8(2)	EEG	0.9678±0.0068	0.9581±0.0095	0.9764±0.0114	0.9731±0.0126	0.9655±0.0073	0.9672±0.0067
	EMG	0.8957±0.0058	0.8914±0.0062	0.8995±0.0093	0.8875±0.0093	0.8893±0.0059	0.8954±0.0057
	ACC	0.9147±0.0064	0.9141±0.0105	0.9152±0.0068	0.9057±0.0072	0.9097±0.0069	0.9147±0.0065
	SC	0.7914±0.0095	0.7637±0.0244	0.8159±0.0157	0.7871±0.0128	0.7748±0.0126	0.7898±0.0099
	EEG+EMG	0.9658±0.0105	0.9537±0.0129	0.9765±0.0115	0.9731±0.0131	0.9632±0.0114	0.9651±0.0106
	EEG+ACC	0.9721±0.0054	0.9657±0.0115	0.9777±0.0086	0.9749±0.0093	0.9702±0.0059	0.9717±0.0055
	EEG+SC	0.9711±0.0072	0.9648±0.0097	0.9767±0.0099	0.9736±0.0109	0.9691±0.0077	0.9707±0.0072
	EMG+ACC	0.9489±0.0052	0.9482±0.0074	0.9495±0.0078	0.9435±0.0083	0.9458±0.0054	0.9488±0.0051
	EMG+SC	0.9382±0.0072	0.9288±0.0121	0.9466±0.0066	0.9394±0.0073	0.9339±0.0079	0.9377±0.0074
	ACC+SC	0.9473±0.0081	0.9434±0.0157	0.9508±0.0102	0.9446±0.0107	0.9439±0.0089	0.9471±0.0084
	EEG+EMG+ACC	0.9726±0.006	0.9659±0.0079	0.9785±0.0089	0.9757±0.0099	0.9707±0.0065	0.9722±0.006
	EEG+EMG+SC	0.9729±0.0077	0.9631±0.0113	0.9816±0.0082	0.9791±0.0093	0.9709±0.0084	0.9724±0.0078
	EEG+ACC+SC	0.9733±0.0061	0.9595±0.0106	0.9855±0.0059	0.9833±0.0068	0.9712±0.0068	0.9725±0.0063
	EMG+ACC+SC	0.9643±0.0073	0.9679±0.0074	0.9611±0.0102	0.9568±0.011	0.9622±0.0076	0.9645±0.0072
	EEG+EMG+ACC+SC	0.9707±0.0073	0.9561±0.0117	0.9838±0.0075	0.9813±0.0085	0.9685±0.008	0.9699±0.0074
9	EEG	0.9909±0.0035	0.8119±0.1224	0.9949±0.0023	0.7959±0.0951	0.7984±0.0929	0.9034±0.0612
	EMG	0.9809±0.0045	0.1657±0.1615	0.9995±0.001	0.67±0.5953	0.2577±0.235	0.5826±0.0811
	ACC	0.975±0.004	0.0857±0.1039	0.9953±0.0027	0.2763±0.4454	0.124±0.1538	0.5405±0.0524
	SC	0.9822±0.0041	0.3452±0.1361	0.9968±0.0031	0.7583±0.2331	0.4546±0.1525	0.671±0.0679
	EEG+EMG	0.9862±0.0043	0.4305±0.1779	0.9989±0.0021	0.8633±0.3393	0.5534±0.2196	0.7147±0.0891
	EEG+ACC	0.9864±0.0044	0.6838±0.1251	0.9933±0.0032	0.7102±0.1442	0.6858±0.1165	0.8386±0.0629
	EEG+SC	0.9883±0.0035	0.5743±0.1482	0.9978±0.0012	0.861±0.106	0.676±0.117	0.786±0.0742
	EMG+ACC	0.976±0.0046	0.2348±0.1704	0.9929±0.004	0.4304±0.2112	0.2902±0.1873	0.6138±0.0847
	EMG+SC	0.9827±0.0044	0.269±0.1453	0.9989±0.0015	0.7803±0.3013	0.3844±0.1674	0.634±0.073
	ACC+SC	0.9829±0.0058	0.5638±0.1304	0.9925±0.0045	0.6499±0.1848	0.5863±0.131	0.7781±0.0658
	EEG+EMG+ACC	0.9861±0.0042	0.4938±0.1577	0.9973±0.0037	0.8395±0.2197	0.5919±0.1456	0.7456±0.0784
	EEG+EMG+SC	0.9826±0.0031	0.8724±0.1164	0.9851±0.0033	0.5904±0.0787	0.6951±0.0713	0.9288±0.0576
	EEG+ACC+SC	0.982±0.0039	0.9019±0.1178	0.9838±0.0052	0.5794±0.0871	0.6973±0.0649	0.9429±0.0572
	EMG+ACC+SC	0.9809±0.0034	0.3914±0.1128	0.9943±0.0033	0.6074±0.215	0.461±0.1285	0.6929±0.056
	EEG+EMG+ACC+SC	0.9826±0.0046	0.7952±0.1073	0.9869±0.0039	0.6013±0.1209	0.6735±0.0899	0.8911±0.054
10	EEG	0.9691±0.0041	0.9646±0.0096	0.9729±0.0048	0.9679±0.0054	0.9662±0.0046	0.9688±0.0044
	EMG	0.8298±0.0072	0.8245±0.0091	0.8343±0.0124	0.8081±0.0114	0.816±0.0072	0.8294±0.007
	ACC	0.8411±0.0068	0.8477±0.0089	0.8355±0.0119	0.8135±0.0105	0.8301±0.0067	0.8416±0.0065
	SC	0.8438±0.0117	0.8116±0.0194	0.871±0.0103	0.8419±0.0112	0.8263±0.0138	0.8413±0.0121
	EEG+EMG	0.966±0.0044	0.9657±0.0097	0.9663±0.0054	0.9604±0.006	0.963±0.0049	0.966±0.0047
	EEG+ACC	0.9706±0.0045	0.9632±0.0051	0.9769±0.0055	0.9725±0.0064	0.9678±0.0049	0.97±0.0044
	EEG+SC	0.9786±0.0044	0.9698±0.0069	0.986±0.0065	0.9833±0.0075	0.9765±0.0048	0.9779±0.0044
	EMG+ACC	0.9038±0.0075	0.8831±0.0089	0.9214±0.009	0.9049±0.0101	0.8937±0.0082	0.9022±0.0075
	EMG+SC	0.9181±0.008	0.8953±0.0124	0.9374±0.0097	0.9237±0.0111	0.9092±0.0089	0.9163±0.0081
	ACC+SC	0.9414±0.0078	0.9273±0.0135	0.9534±0.0124	0.944±0.014	0.9355±0.0085	0.9403±0.0079
	EEG+EMG+ACC	0.9714±0.0036	0.967±0.0066	0.9752±0.0068	0.9706±0.0078	0.9687±0.0039	0.9711±0.0036
	EEG+EMG+SC	0.9768±0.0046	0.9718±0.0102	0.9811±0.0062	0.9776±0.0072	0.9746±0.0051	0.9764±0.0049
	EEG+ACC+SC	0.9792±0.0056	0.972±0.0074	0.9853±0.0072	0.9824±0.0084	0.9772±0.0062	0.9786±0.0056
	EMG+ACC+SC	0.9528±0.0056	0.9414±0.0083	0.9624±0.0064	0.955±0.0073	0.9481±0.0061	0.9519±0.0057

	EEG+EMG+ACC+SC	0.9772±0.0027	0.9672±0.0084	0.9855±0.0039	0.9827±0.0045	0.9749±0.0031	0.9764±0.0031
11	EEG	0.9572±0.0025	0.9264±0.0102	0.971±0.0041	0.9348±0.008	0.9304±0.0042	0.9487±0.0041
	EMG	0.8722±0.0129	0.8193±0.031	0.8957±0.0088	0.7785±0.0189	0.7982±0.0222	0.8575±0.0175
	ACC	0.8519±0.0081	0.8308±0.0241	0.8614±0.0066	0.7281±0.0104	0.7758±0.014	0.8461±0.012
	SC	0.8791±0.0104	0.7228±0.0298	0.9487±0.0108	0.8634±0.0245	0.7864±0.0204	0.8358±0.0146
	EEG+EMG	0.9595±0.0076	0.9387±0.0146	0.9688±0.0066	0.9309±0.014	0.9347±0.0123	0.9538±0.0092
	EEG+ACC	0.9612±0.0086	0.9575±0.0103	0.9628±0.0088	0.9202±0.018	0.9383±0.0134	0.9601±0.0088
	EEG+SC	0.9678±0.0058	0.9459±0.0132	0.9776±0.0048	0.9498±0.0104	0.9477±0.0095	0.9618±0.0075
	EMG+ACC	0.9077±0.0054	0.8827±0.0151	0.9189±0.0067	0.8296±0.0115	0.8551±0.0086	0.9008±0.0072
	EMG+SC	0.9437±0.0079	0.8921±0.0227	0.9667±0.0055	0.9229±0.0121	0.907±0.0138	0.9294±0.0116
	ACC+SC	0.9448±0.0065	0.9255±0.0185	0.9534±0.0045	0.8988±0.0091	0.9118±0.0109	0.9395±0.0095
	EEG+EMG+ACC	0.959±0.0096	0.9324±0.0158	0.9708±0.0093	0.9347±0.0201	0.9334±0.0155	0.9516±0.0108
	EEG+EMG+SC	0.9594±0.0064	0.9032±0.0119	0.9844±0.006	0.9628±0.0139	0.9319±0.0106	0.9438±0.0075
	EEG+ACC+SC	0.9659±0.0058	0.9363±0.0132	0.9791±0.0047	0.9526±0.01	0.9442±0.0095	0.9577±0.0075
	EMG+ACC+SC	0.944±0.0048	0.9399±0.0072	0.9458±0.0061	0.8858±0.0115	0.9119±0.0071	0.9428±0.0047
	EEG+EMG+ACC+SC	0.9506±0.0044	0.9672±0.0072	0.9432±0.0048	0.8841±0.0088	0.9236±0.0066	0.9552±0.0048
12	EEG	0.9767±0.0056	0.9245±0.0354	0.9848±0.0032	0.9062±0.0179	0.9142±0.0218	0.9547±0.0179
	EMG	0.9079±0.0087	0.3925±0.0562	0.9878±0.0038	0.8381±0.0564	0.5313±0.0597	0.6901±0.0285
	ACC	0.813±0.0185	0.6898±0.0413	0.8321±0.0178	0.3904±0.0347	0.4979±0.0366	0.761±0.0259
	SC	0.9453±0.0104	0.6755±0.0474	0.9871±0.0069	0.8915±0.0583	0.7669±0.0446	0.8313±0.0253
	EEG+EMG	0.9776±0.0055	0.9268±0.0325	0.9855±0.004	0.9101±0.0206	0.9176±0.0217	0.9561±0.0164
	EEG+ACC	0.9772±0.005	0.9279±0.0229	0.9848±0.0035	0.9061±0.0195	0.9162±0.0186	0.9564±0.0122
	EEG+SC	0.9873±0.005	0.9664±0.0274	0.9905±0.0032	0.9412±0.0193	0.9533±0.0187	0.9785±0.0141
	EMG+ACC	0.9289±0.0058	0.6728±0.0557	0.9686±0.0055	0.7693±0.0258	0.7167±0.0331	0.8207±0.0262
	EMG+SC	0.956±0.0084	0.734±0.0555	0.9905±0.0041	0.9244±0.0328	0.8167±0.0384	0.8622±0.0279
	ACC+SC	0.9571±0.0092	0.7834±0.0463	0.9841±0.0045	0.8852±0.0317	0.8301±0.0377	0.8837±0.0246
	EEG+EMG+ACC	0.9767±0.0056	0.9042±0.0312	0.988±0.0049	0.9225±0.0267	0.9126±0.021	0.9461±0.0155
	EEG+EMG+SC	0.9792±0.0057	0.8691±0.0417	0.9963±0.0025	0.974±0.0173	0.918±0.0244	0.9327±0.0207
	EEG+ACC+SC	0.9873±0.004	0.9566±0.0257	0.992±0.0036	0.9498±0.0218	0.9529±0.0151	0.9743±0.0125
	EMG+ACC+SC	0.9677±0.0069	0.8815±0.0463	0.981±0.0078	0.8799±0.0401	0.8799±0.0262	0.9313±0.0218
	EEG+EMG+ACC+SC	0.9748±0.0057	0.9781±0.0205	0.9743±0.0082	0.8567±0.0382	0.9128±0.0174	0.9762±0.0082
Average	EEG	0.9629±0.0057	0.9355±0.0409	0.9666±0.0079	0.9349±0.0309	0.9348±0.03	0.9516±0.0207
	EMG	0.8408±0.0093	0.799±0.0556	0.8634±0.0112	0.7776±0.1808	0.7662±0.0746	0.823±0.0286
	ACC	0.8597±0.0105	0.7705±0.0404	0.8328±0.0122	0.7136±0.1354	0.7429±0.05	0.8207±0.0217
	SC	0.8433±0.0113	0.7529±0.0517	0.8712±0.0137	0.7932±0.0751	0.7576±0.0524	0.7993±0.0267
	EEG+EMG	0.9619±0.0067	0.8998±0.0562	0.9672±0.0071	0.9424±0.1031	0.9128±0.0672	0.9321±0.0284
	EEG+ACC	0.964±0.0065	0.9327±0.0404	0.9617±0.0084	0.9221±0.0456	0.9267±0.0368	0.9474±0.0208
	EEG+SC	0.9726±0.0063	0.9253±0.0484	0.9747±0.0066	0.9504±0.034	0.9345±0.0374	0.9484±0.0246
	EMG+ACC	0.9026±0.0066	0.849±0.0564	0.9085±0.0103	0.8211±0.0658	0.8283±0.0585	0.8797±0.0282
	EMG+SC	0.9223±0.007	0.8634±0.0494	0.9409±0.0102	0.8883±0.0923	0.8588±0.0528	0.8966±0.0252
	ACC+SC	0.9291±0.0075	0.8948±0.0444	0.9414±0.0087	0.875±0.0581	0.8803±0.0424	0.9164±0.0228
	EEG+EMG+ACC	0.9618±0.0064	0.906±0.05	0.9677±0.0076	0.9428±0.0677	0.9169±0.0454	0.935±0.0253
	EEG+EMG+SC	0.9608±0.0063	0.9504±0.0395	0.9704±0.0083	0.9185±0.0269	0.9286±0.0248	0.9579±0.0201
	EEG+ACC+SC	0.9706±0.0054	0.952±0.0392	0.9728±0.0065	0.9257±0.0284	0.9349±0.0221	0.9615±0.0194
	EMG+ACC+SC	0.9488±0.0062	0.8924±0.0391	0.9505±0.0096	0.8934±0.0672	0.8892±0.0407	0.9205±0.0198
	EEG+EMG+ACC+SC	0.9676±0.006	0.9367±0.0354	0.9652±0.0063	0.9165±0.0398	0.9267±0.0293	0.9545±0.0181
