# Supplementary material for: Multimodal Data for the Detection of Freezing of Gait in Parkinson’s Disease
Source: Sci Data. 2022 Oct 7;9:606. doi: 10.1038/s41597-022-01713-8 (PMC9546845; doi:10.1038/s41597-022-01713-8)
Supplement: Supplementary file 3 — UPDRS, FOG-Q, MMSE, and MOCA [file 41597_2022_1713_MOESM3_ESM.pdf]

MMSE(in Chinese)

检查前请准备：手表 1 块；铅笔 1 支，白纸 1 张；

评分方法：记录：将患者的回答，记入“记录”栏

评分：答对，记 1 分；答错或拒绝，则记 0 分；将所得分数填写入“评分栏”☐内；

总分计算：各题得分的总和统计为总分，总分范围为 0-30；

	项目		记录	评分
**I 定向力（10 分）**	日期			/
	今年的年份?			☐
	现在是什么季节?			☐
	今天是几月份?			☐
	现在是几号?			☐
	现在是星期几?			☐
	你住在哪个省市？（省、市）			☐
	你住在什么地方？（区、县）			☐
	你住在什么街道？（乡、村）			☐
	这里是什么地方？或医院？（地址、名称）			☐
	我们现在在第几层楼?			☐
**II 记忆力（3 分）**	复述：皮球			☐
	复述：国旗			☐
	复述：树木			☐
**III 注意力和计算力（5 分）**	100-7=?			☐
	-7=?			☐
	-7=?			☐
	-7=?			☐
	-7=?			☐
**IV 回忆能力（3 分）**	回忆：皮球			☐
	回忆：国旗			☐
	回忆：树木			☐
**V 语言能力（9 分）**	**命名能力**	辨认：手表		☐
		辨认：铅笔		☐
	**复述能力**	复述：四十四石只狮子		☐
	**语言理解能力**	按口头指令做动作：右手拿纸		☐
		按口头指令做动作：两手对折		☐
		按口头指令做动作：将放在自己的大腿上		☐
	**阅读理解能力**[1]	按卡片（或纸张）上的指令做动作：闭上你的眼睛		☐
	**书写能力**[2]	写一句完整句子（含主语、动词）		☐

	**结构能力**	按样画图: 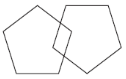			□
			**总分**	□□/30	

[1]**阅读理解能力：**

闭上你的眼睛

[2]**结构能力：画出五边形：**

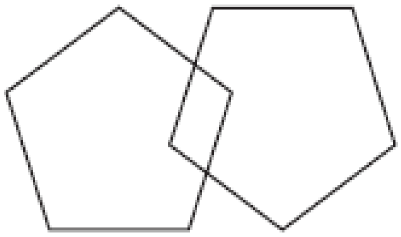

写一句完整的句子：

**临床印象：**
**记分：**<br>**分界值：依据不同教育程度作出的划界分是：**<br>1．文盲组（未受教育）：≤17 分;<br>2．小学组（受教育年限≤6 年）：≤20 分;<br>3．中学（包括中专）：≤22 分;<br>4．大学（包括大专）：≤23 分<br>**结果判定：**总分低于划界分，判定为认知功能受损。

**结论**	
**受试者：文化程度：**□1 文盲 □2 小学 □3 中学 □4 大学	□
**划界分：**□1 17 □2 20 □3 22 □4 23	□
**总　分：**	□□
**是否存在认知功能受损：**□ 0 否 □ 1 是	□

MMSE(in English)

Before testing, please prepare a watch, a pencil, and a piece of white paper.

Scoring Method:

Recording: Record the patient's answers in the "Record" column.

Scoring: One point for a correct answer. Zero points for a wrong answer or refusal. Fill in the scores in the "Scoring" column.

Score Calculating: The sum of the scores of each question is counted as the total score, and the total score ranges from 0-30.

	Projects		Record	Scoring	
**I Orientation (10 points)**	Date				/
	What year is it?				☐
	What season is it?				☐
	What month is it today?				☐
	What date of the month is it?				☐
	What day of the week is it?				☐
	What province or city do you live in? (Province, city)				☐
	Where do you live? (District, County)				☐
	What street do you live in? (Township, village)				☐
	Where are we? Or in which hospital? (address, name)				☐
	Which floor are we on?				☐
**II Memory (3 points)**	Repeat: Pickleball				☐
	Repeat: National Flag				☐
	Repeat: Trees				☐
**III Attention And Computing (5 points)**	100-7=?				☐
	-7=?				☐
	-7=?				☐
	-7=?				☐
	-7=?				☐
**IV Recollection (3 points)**	Recollection: Pickleball				☐
	Recollection: National Flag				☐
	Recollection: Trees				☐
**V Language (9 points)**	**Naming**	Recognition: Watch			☐
		Recognition: Pencil			☐
	**Repetition**	Repeat: Forty-four stone lions			☐
	**Language comprehension**	Perform actions according to verbal instructions: Hold the paper in your right hand.			☐
		Perform actions according to verbal instructions: Fold the paper with both			☐

		hands.			
		Perform actions according to verbal instructions: Place it on your lap.			☐
	**Reading** [1]	Follow the instructions on the card (or paper): Close your eyes.			☐
	**Writting**[2]	Write a complete sentence (including subject and verb)			☐
	**Structural Capability**	Draw a picture by sample : 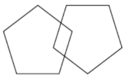			☐
			**Final Score**	☐☐/**30**	

[1] **Reading:**

Close your eyes

[2] **Structural Capability:** Draw a picture by sample**.**

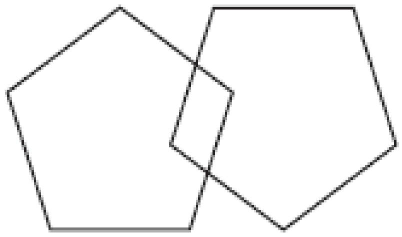

Write a complete sentence:

Clinical impression:

Score:

Cut-offs: The demarcation based on different education levels:

1. Illiterate group (no education): ≤17 points.
2. Primary school group (with ≤ 6 years of education): ≤ 20 points
3. Secondary school (including specialized secondary schools): ≤22 points
4. University (including college): ≤ 23 points

Score result: judged to be cognitively impaired if the total score is below the delineation score.

Results	
**Participants: Education level:**<br>☐1 Illiterate ☐2 Primary school<br>☐3 Secondary school ☐4 University	☐
**Delineation:** ☐1 17 ☐2 20 ☐3 22 ☐4 23	☐
Final Score:	☐☐
Presence of impaired cognitive function: ☐ 0 NO ☐ 1 YES	☐

MoCa(in Chinese)

Montreal Cognitive Assessment (MoCA) Beijing Version
蒙特利尔认知评估北京版

出生日期：
教育水平：
性　　别：
姓名：
检查日期：

得分

视空间与执行功能

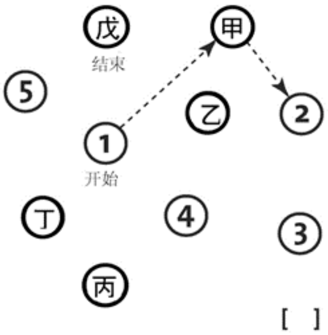

[ ]

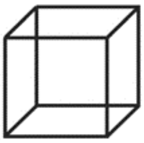

复制立方体

[ ]

画钟表（11点过10分）（3分）

[ ] 轮廓　[ ] 数字　[ ] 指针

__/5

命名

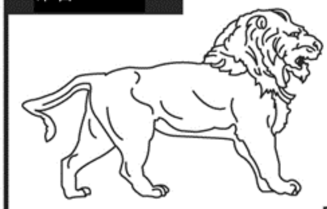

[ ]

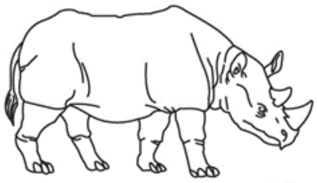

[ ]

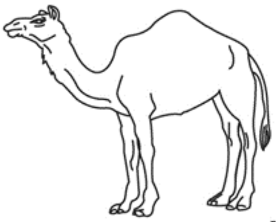

[ ]

__/3

记忆

读出下列词语,而后由患者重复
上述过程重复2次
5分钟后回忆

	面孔	天鹅绒	教堂	菊花	红色
第一次					
第二次					

不计分

注意

读出下列数字,请患者重复（每秒1个）

顺背 [ ] 2 1 8 5 4
倒背 [ ] 7 4 2

__/2

读出下列数字,每当数字1出现时,患者必须用手敲打一下桌面,错误数大于或等于2个不给分

[ ] 5 2 1 3 9 4 1 1 8 0 6 2 1 5 1 9 4 5 1 1 1 4 1 9 0 5 1 1 2

__/1

100连续减7　[ ] 93　[ ] 86　[ ] 79　[ ] 72　[ ] 65

4-5个正确给3分,2-3个正确给2分,1个正确给1分,全都错误为0分

__/3

语言

重复：我只知道今天张亮是来帮过忙的人 [ ]
狗在房间的时候，猫总是躲在沙发下面 [ ]

__/2

流畅性：在1分钟内尽可多的说出动物的名字　[ ] ____ (N ≥ 11 名称)

__/1

抽象

词语相似性:如香蕉-桔子=水果　[ ] 火车-自行车　[ ] 手表-尺子

__/2

延迟回忆

	回忆时不能提示	面孔	天鹅绒	教堂	菊花	红色	仅根据非提示回忆计分
		[ ]	[ ]	[ ]	[ ]	[ ]	
选　项	分类提示						
	多选提示						

__/5

定向

[ ] 日期　[ ] 月份　[ ] 年代　[ ] 星期几　[ ] 地点　[ ] 城市

__/6

总分 __/30

© Z.Nasreddine MD Version November 7, 2004

Beijing version 26 August, 2006 translated by Wei Wang & Hengge Xie

www.mocatest.org

MoCa(in English)

NAME :
Education : Date of birth :
Sex : DATE :

MONTREAL COGNITIVE ASSESSMENT (MOCA)

Version 7.1 Original Version

VISUOSPATIAL / EXECUTIVE			POINTS
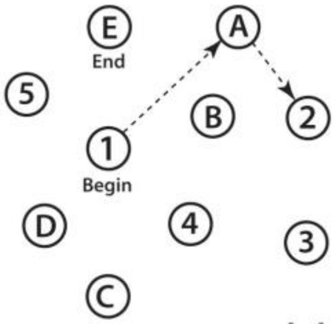 [ ]	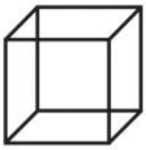 Copy cube [ ]	Draw CLOCK (Ten past eleven) (3 points) [ ] Contour [ ] Numbers [ ] Hands	___/5

NAMING

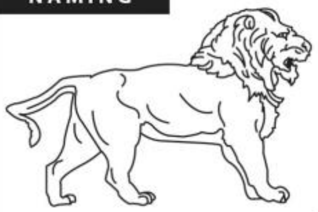
[ ]

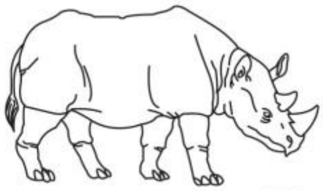
[ ]

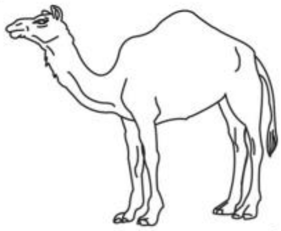
[ ]

___/3

MEMORY Read list of words, subject must repeat them. Do 2 trials, even if 1st trial is successful. Do a recall after 5 minutes.

	FACE	VELVET	CHURCH	DAISY	RED
1st trial					
2nd trial					

No points

ATTENTION Read list of digits (1 digit/ sec.). Subject has to repeat them in the forward order [ ] 2 1 8 5 4
Subject has to repeat them in the backward order [ ] 7 4 2 ___/2

Read list of letters. The subject must tap with his hand at each letter A. No points if ≥ 2 errors
[ ] F B A C M N A A J K L B A F A K D E A A A J A M O F A A B ___/1

Serial 7 subtraction starting at 100 [ ] 93 [ ] 86 [ ] 79 [ ] 72 [ ] 65
4 or 5 correct subtractions: **3 pts**, 2 or 3 correct: **2 pts**, 1 correct: **1 pt**, 0 correct: **0 pt** ___/3

LANGUAGE Repeat : I only know that John is the one to help today. [ ]
The cat always hid under the couch when dogs were in the room. [ ] ___/2

Fluency / Name maximum number of words in one minute that begin with the letter F [ ] _____ (N ≥ 11 words) ___/1

ABSTRACTION Similarity between e.g. banana - orange = fruit [ ] train – bicycle [ ] watch - ruler ___/2

DELAYED RECALL		FACE	VELVET	CHURCH	DAISY	RED		
	Has to recall words **WITH NO CUE**	[ ]	[ ]	[ ]	[ ]	[ ]	Points for UNCUED recall only	___/5
Optional	Category cue							
	Multiple choice cue							

ORIENTATION [ ] Date [ ] Month [ ] Year [ ] Day [ ] Place [ ] City ___/6

© Z.Nasreddine MD www.mocatest.org Normal ≥26 / 30 TOTAL ___/30

Administered by: ______________________________ Add 1 point if ≤ 12 yr edu

UPDRS(in Chinese&English)

________________	___ ___ ___	__ __ - __ __ - __ __ __ __ (mm-dd-yyyy)	___ ___ ___
Patient Name or Subject ID	Site ID	Assessment Date	Investigator's Initials

MDS UPDRS Score Sheet

1.A	Source of information	☐ Patient ☐ Caregiver ☐ Patient + Caregiver	3.3b	Rigidity– RUE 强直-右上肢	
			3.3c	Rigidity– LUE 强直-左上肢	
**Part I**	日常生活非运动症状体验		3.3d	Rigidity– RLE 强直-右下肢	
1.1	Cognitive impairment 认知损害		3.3e	Rigidity– LLE 强直-左下肢	
1.2	Hallucinations and psychosis 幻觉和精神症状		3.4a	Finger tapping– Right hand 手指拍打-右	
1.3	Depressed mood 抑郁情绪		3.4b	Finger tapping– Left hand 手指拍打-左	
1.4	Anxious mood 焦虑情绪		3.5a	Hand movements– Right hand 手掌运动-右	
1.5	Apathy 淡漠		3.5b	Hand movements– Left hand 手掌运动-左	
1.6	Features of DDS 多巴胺失调的特征		3.6a	Pronation- supination movements– Right hand 右前臂回旋	
1.6a	Who is filling out questionnaire	☐ Patient ☐ Caregiver ☐ Patient + Caregiver	3.6b	Pronation- supination movements– Left hand 左	
			3.7a	Toe tapping–Right foot 脚趾拍地-右	
1.7	Sleep problems 睡眠问题		3.7b	Toe tapping– Left foot 脚趾拍地-左	
1.8	Daytime sleepiness 白日嗜睡		3.8a	Leg agility– Right leg 腿灵敏性-右	
1.9	Pain and other sensations 疼痛和其它感觉		3.8b	Leg agility– Left leg 腿灵敏性-左	
1.10	Urinary problems 排尿问题		3.9	Arising from chair 起立	
1.11	Constipation problems 便秘问题		3.10	Gait 步态	
1.12	Light headedness on standing 站起时头晕		3.11	Freezing of gait 冻结步态	
1.13	Fatigue 疲劳感		3.12	Postural stability 姿势平稳度	
**Part II**	日常生活运动症状体验		3.13	Posture 姿势	
2.1	Speech 言语		3.14	Global spontaneity of movement 全身自发性动作评估	
2.2	Saliva and drooling 唾液分泌与流口水		3.15a	Postural tremor– Right hand 双手姿势性震颤-右	
2.3	Chewing and swallowing 咀嚼与吞咽		3.15b	Postural tremor– Left hand 双手姿势性震颤-左	
2.4	Eating tasks 进食能力		3.16a	Kinetic tremor– Right hand 双手动作性震颤-右	
2.5	Dressing 穿衣		3.16b	Kinetic tremor– Left hand 双手动作性震颤-左	
2.6	Hygiene 卫生清洁		3.17a	Rest tremor amplitude– RUE 静止性震颤幅度-右上肢	
2.7	Handwriting 写字		3.17b	Rest tremor amplitude– LUE 静止性震颤幅度-左上肢	
2.8	Doing hobbies and other activities 嗜好和其它活动		3.17c	Rest tremor amplitude– RLE 静止性震颤幅度-右下肢	
2.9	Turning in bed 翻身		3.17d	Rest tremor amplitude– LLE 静止性震颤幅度-左下肢	
2.10	Tremor 震颤		3.17e	Rest tremor amplitude– Lip/jaw 静止性震颤幅度-嘴唇	
2.11	Getting out of bed 起床、离开车或从较低的椅子起身		3.18	Constancy of rest 静止性震颤持续性	
2.12	Walking and balance 走路与平衡			Were dyskinesias presen 异动症是否出现	☐ No ☐ Yes
2.13	Freezing 冻结			Did these movements interfere with ratings?	☐ No ☐ Yes
3a	Is the patient on medication?	☐ No ☐ Yes		Hoehn and Yahr Stage	
3b	Patient's clinical state	☐ Off ☐ On	**Part IV**	运动并发症	
3c	Is the patient on Levodopa?	☐ No ☐ Yes	4.1	Time spent with dyskinesias 出现异动症的时间	
3.C1	If yes, minutes since last dose:		4.2	Functional impact of dyskinesias 对生活的影响	
**Part III**	运动功能检查		4.3	Time spent in the OFF state 发生关的时间	
3.1	Speech 言语		4.4	Functional impact of fluctuations 对生活的影响	
3.2	Facial expression 面部表情		4.5	Complexity of motor fluctuations 药效波动的复杂性	
3.3a	Rigidity– Neck 强直-脖子		4.6	Painful OFF-state dystonia 痛性关期肌张力障碍	

July 1, 2008

Copyright © 2008 Movement Disorder Society. All rights reserved.
This chart may not be copied, distributed or otherwise used in whole or in part without prior written consent of the Movement Disorder Society

FOGQ(in Chinese)

冻结步态问卷（FOGQ)		评分
1 在你状态最差的时候走路	(0) 正常<br>(1) 基本正常—稍微缓慢<br>(2) 缓慢但是完全独立<br>(3) 需要帮助或是助行器<br>(4) 不能行	
2 你的步态困难影响你的日常活动和独立做事吗?	(0) 完全不<br>(1) 有点儿<br>(2) 中等的<br>(3) 严重地<br>(4) 不能行走	
3 你在行走时、转身或启动行走时有没有双脚黏住地面的感觉（冻结）?	(0) 从来没有<br>(1) 极少（约 1 次/月）<br>(2) 较少（约 1 次/周）<br>(3) 经常（约 1 次/天）<br>(4) 行走时总发生	
4 你最长的一次冻结步态是多长时间?	(0) 从未发生<br>(1) 1-2 秒<br>(2) 3-10 秒<br>(3) 11-30 秒<br>(4) 超过 30 秒不能行走	
5 你发生一次典型的启动犹豫是多长时间(开始第一步时冻结）?	(0) 无<br>(1) 超过 1 秒启动行走<br>(2) 超过 3 秒启动行走<br>(3) 超过 10 秒启动行走<br>(4) 超过 30 秒启动行走	
6 你发生一次典型的转身犹豫是多长时间(转身冻结）?	(0) 无<br>(1) 1-2 秒完成转身<br>(2) 3-10 秒完成转身<br>(3) 11-30 秒完成转身<br>(4) 超过 30 秒无法转身	

FOGQ(in English)

**Freezing Of Gait Questionnaire （FOGQ)**		Score
1 Walk when you're in the worst status	(0) Normal<br>(1) Mostly normal, a bit slow<br>(2) Slow but completely independent<br>(3) Need help or a walking assistant<br>(4) Unable to walk	
2 Does your gait problem affect your daily activities and individual work?	(0) Not at all<br>(1) A little bit<br>(2) Intermediate<br>(3) Badly<br>(4) Unable to walk	
3 Do you feel like your feet are glued to the floor when you walk, turn around, or start walking?	(0) Never<br>(1) Hardly ever (About once a month)<br>(2) Sometimes (About once a week)<br>(3) Frequently (About once a day)<br>(4) It happens all the time while walking	
4 What's the longest frozen gait you've ever had?	(0) Never<br>(1) 1-2 seconds<br>(2) 3-10 seconds<br>(3) 11-30 seconds<br>(4) More than 30 seconds	
5 How long do you experience a typical startup hesitation (freeze on the first step)?	(0) Never<br>(1) More than 1 second<br>(2) More than 3 seconds<br>(3) More than 10 seconds<br>(4) More than 30 seconds	
6 How long do you experience a typical turning hesitation (freeze when turning around)?	(0) Never<br>(1) 1-2 seconds<br>(2) 3-10 seconds<br>(3) 11-30 seconds<br>(4) More than 30 seconds	
