# Supplementary material for: Multimodal Data for the Detection of Freezing of Gait in Parkinson’s Disease
Source: Sci Data. 2022 Oct 7;9:606. doi: 10.1038/s41597-022-01713-8 (PMC9546845; doi:10.1038/s41597-022-01713-8)
Supplement: Supplementary file 4 — DATA QUALITY CHECK REPORT [file 41597_2022_1713_MOESM4_ESM.pdf]

DATA QUALITY CHECK REPORT

We use three methods to demonstrate the quality of the data. Firstly, we generate a data summary report containing useful statistical information for each column in a data table. Secondly, we calculate Noise Proportion and the SNR(signal-to-noise ratio). Thirdly, we calculate EEG Quality Index. This report contains the results of these three parts: "Data Summary Report", "Noise Proportion & SNR", and "EEG Quality Index".

Data Summary Report

In this part, we generate a data summary report containing useful statistical information for each column. Column 'type' shows data types including 'key', 'str', 'date', 'numeric'. Column 'min' shows the minimum value of the channel. Column 'median' shows the mid-value of the channel. Column 'max' shows the maximum value of the channel. Column 'std' shows the standard deviation of the channel.

Data Summary Report (Patient ID:01)					
column	type	min	median	max	std
TIME	date				
FP1	numeric	-91.885	0.00735	152.0935	13.41605304
FP2	numeric	-96.3312	0.0351	153.0949	12.1412642
F3	numeric	-79.4359	-0.0152	135.7594	11.61855707
F4	numeric	-101.0593	0.0401	125.3928	11.66616277
C3	numeric	-79.1242	0.00235	110.0145	11.07370299
C4	numeric	-100.6773	-0.00825	118.8376	10.78563398
P3	numeric	-79.8571	-0.0768	85.1997	11.66000375
P4	numeric	-90.5781	0.024	101.5372	12.4878892
O1	numeric	-105.5042	0.1263	117.1223	16.53865461
O2	numeric	-130.1996	0.2919	119.9336	16.9524604
F7	numeric	-76.1704	-0.0395	81.0312	9.808166838
F8	numeric	-112.0897	0.04685	64.6379	8.351265581
P7	numeric	-93.9151	-0.2059	86.3731	13.43818094
P8	numeric	-115.0615	0.0287	83.5673	11.56272679
Fz	numeric	-91.6799	0.03365	110.7002	12.34374202
Cz	numeric	-100.6736	-0.0142	95.8678	12.25580855
Pz	numeric	-108.4387	-0.19195	82.542	13.38658537
FC1	numeric	-80.6058	0.0238	106.4885	11.58861984
FC2	numeric	-98.066	-0.0206	102.546	12.13259247
CP1	numeric	-136.7962	-0.29245	143.3187	16.92664084
CP2	numeric	-103.6983	-0.0538	94.4427	12.77147406

FC5	numeric	-85.7792	0.02045	86.5899	10.22662291
FC6	numeric	-102.3278	-0.0164	148.4609	9.780603537
CP5	numeric	-94.9241	-0.08335	97.6425	10.96768528
CP6	numeric	-118.9625	-0.0041	110.8193	9.671782059
EMG1	numeric	-6050	355	6117	2828.662811
EMG2	numeric	-4060.5	-54.5	5477.5	923.6777225
IO	numeric	-391.5	-0.5	412.5	47.65093748
EMG3	numeric	-5212	-926.5	6960.5	2629.968786
EMG4	numeric	-5962.5	85.5	6218.5	3056.224003
LShankACCX	numeric	-36396.8793	8368.033036	38698.41869	3996.134396
LShankACCY	numeric	-40896.0022	-515.249042	41370.34091	5208.77735
LShankACCZ	numeric	-22963	-1626.80136	35028.10252	2510.707123
LShankGYROX	numeric	-10334.5504	-134	9396	1240.874523
LShankGYROY	numeric	-8191.90386	-9.3982136	12110.33593	609.5455933
LShankGYROZ	numeric	-10846	-10	9582.815807	2602.300528
NC	numeric	75.73878331	77	78	0.219353308
RShankACCX	numeric	0	0	0	0
RShankACCY	numeric	0	0	0	0
RShankACCZ	numeric	0	0	0	0
RShankGYROX	numeric	0	0	0	0
RShankGYROY	numeric	0	0	0	0
RShankGYROZ	numeric	0	0	0	0
NC.1	numeric	0	0	0	0
WaistACCX	numeric	176.0821909	8053.665371	23048.62052	932.7320135
WaistACCY	numeric	-14541.2089	315	13874	978.7077403
WaistACCZ	numeric	-8510.59622	1319.998235	8768.860879	1088.561871

WaistGYROX	numeric	-3556.03919	-25.0795766	4082.949661	605.3671823
WaistGYROY	numeric	-9608.23248	54.05350986	3041.921238	324.2850793
WaistGYROZ	numeric	-2197.93592	28.1064551	2466.626157	264.2256899
NC.2	numeric	51.80707561	53	54.32935494	0.095180735
ArmACCX	numeric	-7771.26749	7565.670259	18702.65945	1248.014358
ArmACCY	numeric	-12762.8578	-1324	25965	2762.522581
ArmACCZ	numeric	-22394.5762	769.2168005	27700	1519.9314
ArmGYROX	numeric	-16969.6784	46.72652136	18749	770.6079212
ArmGYROY	numeric	-5476.96296	38	9079.715558	412.1732907
ArmGYROZ	numeric	-4676.54305	19.68004186	6131.977916	458.9856378
SC	numeric	1320.881121	1789	1884.110225	57.57877338
Label	numeric	0	0	1	0.475460089

Data Summary Report (Patient ID:02)					
column	type	min	median	max	std
TIME	date				
FP1	numeric	-103.5919	-0.0091	107.1869	13.64910014
FP2	numeric	-124.925	-0.1197	80.3895	12.04719556
F3	numeric	-106.5385	-0.0998	120.9645	12.9749936
F4	numeric	-109.2813	-0.0985	123.0271	12.68037185
C3	numeric	-75.6473	-0.0423	76.8944	12.1355526
C4	numeric	-73.6014	-0.05945	72.0625	11.7031114
P3	numeric	-73.7694	0.0223	77.9897	11.81227274
P4	numeric	-80.5846	-0.0751	81.9202	12.23152036
O1	numeric	-70.8122	-0.0006	65.075	12.15512676
O2	numeric	-74.2584	0.02055	73.8508	12.36728745
F7	numeric	-111.89	-0.2971	261.997	19.19384135
F8	numeric	-64.3154	-0.01775	60.6907	10.17331491
P7	numeric	-65.0144	0.0209	92.7462	12.05191378
P8	numeric	-66.4949	-0.0827	70.1899	11.16215915
Fz	numeric	-68.6294	-0.18705	85.3899	13.24412864
Cz	numeric	-73.2543	-0.1743	71.8246	13.52302541
Pz	numeric	-75.1778	-0.1487	80.1616	12.61641653
FC1	numeric	-82.6611	-0.08945	82.911	12.32296668
FC2	numeric	-77.8118	-0.1114	88.7413	12.27990102
CP1	numeric	-75.7145	-0.0749	69.0742	12.2482814
CP2	numeric	-74.6327	-0.1153	102.7364	12.69424097

FC5	numeric	-285.5168	2.47635	207.4749	57.33623173
FC6	numeric	-202.6159	0.2775	94.0999	13.65994491
CP5	numeric	-78.9169	-0.014	81.9065	13.57693781
CP6	numeric	-74.0148	-0.1143	65.0878	11.38822826
EMG1	numeric	-5874	-920	6695	3181.948299
EMG2	numeric	-6035.5	-224.5	6010.5	3078.103645
IO	numeric	-589.5	2	268.5	47.31732051
EMG3	numeric	-3654	12.5	3615	1299.041706
EMG4	numeric	-6763	-874.5	6495.5	3287.193026
LShankACCX	numeric	0	0	0	0
LShankACCY	numeric	0	0	0	0
LShankACCZ	numeric	0	0	0	0
LShankGYROX	numeric	0	0	0	0
LShankGYROY	numeric	0	0	0	0
LShankGYROZ	numeric	0	0	0	0
NC	numeric	0	0	0	0
RShankACCX	numeric	-4687.61212	8310.101131	35292.13143	2140.206838
RShankACCY	numeric	-12284.6835	-937.478494	21623.02351	1827.925948
RShankACCZ	numeric	-41107.1422	-1439	31360.00766	3473.655734
RShankGYROX	numeric	-14002.1804	-330.883715	9607.944638	1636.464668
RShankGYROY	numeric	-9468	-732	9932.916527	2920.051613
RShankGYROZ	numeric	-3802.7825	4.442435962	3817.927458	584.1806497
NC.1	numeric	75.73666554	77	78	0.293518827
WaistACCX	numeric	4370.139346	8016.006819	17988.08071	1019.856584
WaistACCY	numeric	-6145.1221	-1123.52542	3451.851133	976.6976091
WaistACCZ	numeric	-3181.05699	549.159868	6375	973.0831693
WaistGYROX	numeric	-3705.70917	-53	4419.287137	808.9128649
WaistGYROY	numeric	-5177.19738	66	3610.461519	425.1246297
WaistGYROZ	numeric	-1472	17.25001906	1486.285842	311.2834477
NC.2	numeric	51.66084972	53	1010	46.90293955
ArmACCX	numeric	0	0	0	0
ArmACCY	numeric	0	0	0	0
ArmACCZ	numeric	0	0	0	0
ArmGYROX	numeric	0	0	0	0
ArmGYROY	numeric	0	0	0	0
ArmGYROZ	numeric	0	0	0	0

SC	numeric	0	0	0	0
Label	numeric	0	0	1	0.074533884
Data Summary Report (Patient ID:03)					
column	type	min	median	max	std
TIME	date				
FP1	numeric	-103.2655	0.11855	98.6956	15.26843996
FP2	numeric	-102.0377	0.1071	94.155	14.39254736
F3	numeric	-100.2001	0.22465	89.2764	14.56818819
F4	numeric	-110.7329	0.14345	104.277	13.87040984
C3	numeric	-92.9945	0.1097	92.5252	13.25135508
C4	numeric	-87.7989	0.0953	83.0712	12.62403907
P3	numeric	-88.082	0.15765	96.5045	12.55241733
P4	numeric	-91.0453	0.0999	89.8932	12.39734411
O1	numeric	-137.6721	0.1781	117.7145	14.41739279
O2	numeric	-253.6699	0.0785	392.5437	17.00673077
F7	numeric	-92.6347	0.1827	91.2868	12.75291789
F8	numeric	-87.3583	0.1361	84.1746	11.46594876
P7	numeric	-103.1689	0.32845	90.5676	12.705332
P8	numeric	-83.1889	0.1977	98.1934	10.05651227
Fz	numeric	-94.8929	0.1753	83.7373	14.48930736
Cz	numeric	-93.3211	0.142	89.3739	13.73380489
Pz	numeric	-101.0541	0.06435	108.9709	16.10647802
FC1	numeric	-99.2603	0.0936	91.5924	14.24203781
FC2	numeric	-96.8133	0.1302	88.9621	13.5857506
CP1	numeric	-90.6602	0.1565	95.2795	13.12445256
CP2	numeric	-96.2173	0.0678	100.8971	12.85784034
FC5	numeric	-123.1934	0.1185	124.4073	16.18662395
FC6	numeric	-99.3009	0.0804	99.1298	13.21160981
CP5	numeric	-87.1163	0.2009	88.6055	11.14348147
CP6	numeric	-87.6413	0.1512	94.0926	10.5658077
EMG1	numeric	-5961	52	6049	2876.912537
EMG2	numeric	-6996	136.5	6913	2698.474969
IO	numeric	-403	7	290	61.28253099
EMG3	numeric	-6795.5	101.5	5805.5	2625.033903
EMG4	numeric	-7223.5	408	5986	2884.595914
LShankACCX	numeric	-5693.1129	7868	30165.71018	1665.864084
LShankACCY	numeric	-38166.8844	-2910.50925	26376.49489	2362.139905
LShankACCZ	numeric	-12316	-1721.47269	21929.75284	1517.68632
LShankGYROX	numeric	-6109	-74.2451245	7460	724.2142627
LShankGYROY	numeric	-5666.65724	-17.4419861	3876.313742	754.7992854

LShankGYROZ	numeric	-6317.2312	58.0888344	3340	872.670738
NC	numeric	56.77176331	77	2453.756963	28.06731878
RShankACCX	numeric	0	0	0	0
RShankACCY	numeric	0	0	0	0
RShankACCZ	numeric	0	0	0	0
RShankGYROX	numeric	0	0	0	0
RShankGYROY	numeric	0	0	0	0
RShankGYROZ	numeric	0	0	0	0
NC.1	numeric	0	0	0	0
WaistACCX	numeric	3847	8167.622929	15336.13776	769.9613886
WaistACCY	numeric	-2908.13999	694	6089.332804	640.7306012
WaistACCZ	numeric	-5694.76532	-1043	4771.199932	602.3498797
WaistGYROX	numeric	-2137	19.03055189	2628.286317	434.9875758
WaistGYROY	numeric	-3202.2395	19.69688198	4041.303799	240.6642588
WaistGYROZ	numeric	-1259.45576	4.989484746	1148	219.5558422
NC.2	numeric	2558	2563	2570.084579	1.593234511
ArmACCX	numeric	-4034.43037	6311.510247	11151.7939	1410.333338
ArmACCY	numeric	-161	4111.504307	11424.41914	1321.937951
ArmACCZ	numeric	-4881.62095	2860.057543	8260.804264	985.3624583
ArmGYROX	numeric	-2796.33517	46	4259.695095	455.0870256
ArmGYROY	numeric	-4600.48052	48.93194917	5589.014438	305.9814016
ArmGYROZ	numeric	-3784.60271	10	3391	257.0140992
SC	numeric	602.8923996	817.0007431	1097.146782	72.89693424
Label	numeric	0	1	1	0.358325574
Data Summary Report (Patient ID:04)					
column	type	min	median	max	std
TIME	date				
FP1	numeric	-78.2494	-0.03305	124.0571	12.24739564
FP2	numeric	-85.8896	-0.0776	139.273	12.83971877
F3	numeric	-73.0173	-0.032	109.5058	10.88731904
F4	numeric	-73.302	-0.00165	102.6454	10.81638406
C3	numeric	-64.828	-0.11015	93.0539	10.62722729
C4	numeric	-65.7965	-0.0846	82.2749	10.10427308
P3	numeric	-72.8247	-0.26925	78.5097	11.26694856
P4	numeric	-70.9907	-0.1168	88.9885	11.83565782
O1	numeric	-104.047	-0.4455	136.372	16.50230575
O2	numeric	-92.7719	-0.35335	115.9844	14.95754308
F7	numeric	-61.7504	0.0431	103.5231	9.438693929

F8	numeric	-58.8729	0.05215	83.8482	8.53699922
P7	numeric	-69.2276	-0.27675	103.3089	12.16108471
P8	numeric	-63.6458	-0.02615	76.3719	8.048548095
Fz	numeric	-70.3463	-0.0841	98.9436	12.33565201
Cz	numeric	-63.9641	-0.1778	81.94	11.26851518
Pz	numeric	-69.2021	-0.2749	86.4156	10.98346352
FC1	numeric	-69.153	-0.0779	93.2396	11.19700379
FC2	numeric	-68.8076	-0.0415	86.4826	10.79527
CP1	numeric	-80.7327	-0.26085	101.014	13.51012159
CP2	numeric	-68.0637	-0.2436	93.7349	11.15860829
FC5	numeric	-65.4126	0.0198	97.1925	9.433218369
FC6	numeric	-60.5674	0.0513	77.592	8.80082913
CP5	numeric	-68.6982	-0.1568	90.4376	10.05740454
CP6	numeric	-66.6079	-0.08865	67.259	8.906370581
EMG1	numeric	-5833.5	57	5272.5	2251.375996
EMG2	numeric	-5186.5	-4.5	4864.5	1834.933323
IO	numeric	-476.5	0	330.5	45.04233367
EMG3	numeric	-827.5	-11	1088.5	122.833672
EMG4	numeric	-5604.5	14	5853	2667.446271
LShankACCX	numeric	0	0	0	0
LShankACCY	numeric	0	0	0	0
LShankACCZ	numeric	0	0	0	0
LShankGYROX	numeric	0	0	0	0
LShankGYROY	numeric	0	0	0	0
LShankGYROZ	numeric	0	0	0	0
NC	numeric	0	0	0	0
RShankACCX	numeric	-5719.45173	8323	33959.15562	2308.823191
RShankACCY	numeric	-15603.8083	-1071.19546	21102	1968.882513
RShankACCZ	numeric	-37646.4611	-1231.93617	20634.43434	3707.194768
RShankGYROX	numeric	-10535.0393	-163	11846.8023	1729.902555
RShankGYROY	numeric	-8107.20468	-352.056153	12343.70401	3374.509976
RShankGYROZ	numeric	-5076.79833	60.2064833	3297.173987	589.2632044
NC.1	numeric	75.738765	77	78	0.260173008
WaistACCX	numeric	0	0	0	0
WaistACCY	numeric	0	0	0	0
WaistACCZ	numeric	0	0	0	0
WaistGYROX	numeric	0	0	0	0

WaistGYRO Y	numeric	0	0	0	0
WaistGYRO Z	numeric	0	0	0	0
NC.2	numeric	0	0	0	0
ArmACCX	numeric	2771	7300	11898.73455	1266.789539
ArmACCY	numeric	-339.896212	3968.109918	10077.37372	1590.107941
ArmACCZ	numeric	-3639.66645	-161.650598	4798.911256	1079.443563
ArmGYROX	numeric	-3638.87344	105.903033	3594.738083	766.3558312
ArmGYROY	numeric	-2176.64423	59.05053516	2727	532.7486648
ArmGYROZ	numeric	-1876.93039	20.57306931	1862.397219	310.3784876
SC	numeric	923.7782339	1081.000747	1148.216324	54.92811006
Label	numeric	0	0	1	0.404378181
Data Summary Report (Patient ID:05)					
column	type	min	median	max	std
TIME	date				
FP1	numeric	-273.963	4.76215	297.2603	57.96571749
FP2	numeric	-139.0936	-1.23045	184.2444	28.66332591
F3	numeric	-136.4498	-1.6931	135.2088	35.70613873
F4	numeric	-134.925	-1.97405	135.9692	35.72854057
C3	numeric	-131.9449	-1.9008	133.5053	33.78801761
C4	numeric	-127.1585	-1.86	131.1551	32.15523092
P3	numeric	-130.8556	-1.9069	131.0705	34.11572045
P4	numeric	-128.531	-1.7191	126.4307	31.40812983
O1	numeric	-181.9719	-1.37335	219.2224	36.42665138
O2	numeric	-146.3465	0.04625	217.6439	31.31685413
F7	numeric	-387.27	1.72335	393.8465	65.74129866
F8	numeric	-213.3751	1.6861	292.8329	39.69825956
P7	numeric	-144.0109	0.053	271.3844	26.8174398
P8	numeric	-136.2836	-0.3698	136.6249	26.47039136
Fz	numeric	-132.1853	-1.7219	146.0338	37.3589925
Cz	numeric	-130.1243	-1.8759	140.7253	37.31407423
Pz	numeric	-126.2947	-1.35295	140.2471	31.61520031
FC1	numeric	-129.478	-1.77295	139.1188	37.16472461
FC2	numeric	-134.1497	-1.7462	139.4045	36.77272291
CP1	numeric	-213.9416	-1.64925	227.6492	40.98041139
CP2	numeric	-127.675	-1.9335	138.127	36.64883946
FC5	numeric	-145.1102	0.8413	102.7217	25.2888223
FC6	numeric	-117.1595	-0.3222	91.1804	19.73623149
CP5	numeric	-131.8497	-0.62375	141.4614	21.62347426
CP6	numeric	-161.9118	0.10555	185.2909	27.8729841
EMG1	numeric	-5256.5	10.5	6009.5	2267.58666
EMG2	numeric	-4975.5	-109.5	5581.5	2109.538085
IO	numeric	-522	0	498.5	89.55523206

EMG3	numeric	-3254.5	-96	4428.5	805.94413
EMG4	numeric	-4708	-44.25	4683	1563.729724
LShankACCX	numeric	-6562.82878	8326	35834.92152	2084.73262
LShankACCY	numeric	-39568.3223	29.36470898	35270.56476	2312.129401
LShankACCZ	numeric	-40962.7265	-1894.40735	35429.56285	3626.179394
LShankGYROX	numeric	-15897.8734	-29	16052.81713	1911.625271
LShankGYROY	numeric	-9578.08634	-507.525563	14739	3399.983555
LShankGYROZ	numeric	-8034.89064	71	5975	1331.256075
NC	numeric	75.73681862	77	78	0.288399402
RShankACCX	numeric	0	0	0	0
RShankACCY	numeric	0	0	0	0
RShankACCZ	numeric	0	0	0	0
RShankGYROX	numeric	0	0	0	0
RShankGYROY	numeric	0	0	0	0
RShankGYROZ	numeric	0	0	0	0
NC.1	numeric	0	0	0	0
WaistACCX	numeric	0	0	0	0
WaistACCY	numeric	0	0	0	0
WaistACCZ	numeric	0	0	0	0
WaistGYROX	numeric	0	0	0	0
WaistGYROY	numeric	0	0	0	0
WaistGYROZ	numeric	0	0	0	0
NC.2	numeric	0	0	0	0
ArmACCX	numeric	-11742	7800	22726.96535	1357.414299
ArmACCY	numeric	-32399	2197.729004	19429.82346	1473.758762
ArmACCZ	numeric	-31952	196.7150292	25743.66112	1435.891121
ArmGYROX	numeric	-21389.4254	59.64894306	22544	1103.454932
ArmGYROY	numeric	-15421.4286	45.36151132	11851.16933	476.4659759
ArmGYROZ	numeric	-4650	24.58613922	3563.296056	543.3859712
SC	numeric	786.8923997	878.1867973	1116.085158	29.34066394
Label	numeric	0	0	0	0
Data Summary Report (Patient ID:06)					
column	type	min	median	max	std
TIME	date				
FP1	numeric	-343.4564	-0.5051	304.7481	35.82828728
FP2	numeric	-322.0232	-0.69415	301.2773	35.03888207

F3	numeric	-294.1884	-0.55045	348.4841	33.20114641
F4	numeric	-334.2156	-0.7071	320.4286	35.93995983
C3	numeric	-289.1635	-0.39855	312.6903	31.90919102
C4	numeric	-330.0471	-0.71515	305.5779	35.60581365
P3	numeric	-308.5544	-0.1532	258.9401	33.37133593
P4	numeric	-348.0692	-0.87865	292.3175	37.0454066
O1	numeric	-389.7856	-0.29545	310.2475	39.92146
O2	numeric	-383.12	-0.8864	300.4833	40.61799546
F7	numeric	-292.1003	-0.47655	260.3987	31.76735347
F8	numeric	-323.9304	-0.75755	283.0661	35.91064906
P7	numeric	-298.1994	0.88395	150.7999	26.57675038
P8	numeric	-342.6004	-0.68405	281.8042	39.20869429
Fz	numeric	-330.3021	-0.5685	307.1311	35.19852043
Cz	numeric	-326.1961	-0.5934	291.8718	34.86052737
Pz	numeric	-322.0077	-0.51295	283.5014	35.15837412
FC1	numeric	-317.911	-0.5666	317.4085	34.10009986
FC2	numeric	-336.4811	-0.6427	307.2713	35.45710207
CP1	numeric	-310.0744	-0.4447	292.9143	34.29084976
CP2	numeric	-324.768	-0.6112	294.2628	35.4489058
FC5	numeric	-265.147	-0.32025	254.1865	30.41772666
FC6	numeric	-326.3538	-0.7451	281.686	36.38542872
CP5	numeric	-219.3744	0.19965	211.2555	25.73883005
CP6	numeric	-333.4464	-0.7894	306.5688	37.07516158
EMG1	numeric	-7222.5	-156.5	6539.5	2978.133472
EMG2	numeric	-3588	-26.5	3464.5	439.6500697
IO	numeric	-746.5	0	425	51.73029799
EMG3	numeric	-5675	18.5	5462.5	1649.442103
EMG4	numeric	-6757.5	-713	6712.5	3132.294963
LShankACCX	numeric	-8742	7920.359713	32898.08164	1377.63216
LShankACCY	numeric	-28189.726	-448.424624	13820.05731	1404.355954
LShankACCZ	numeric	-35564.6695	-3373.02372	15154	2267.999109
LShankGYROX	numeric	-13120.8883	-66.8113912	8288.10497	1014.884465
LShankGYROY	numeric	-5137.61601	-340.665069	9388	1773.136466
LShankGYROZ	numeric	-5018.20516	64.48407674	3608.443945	883.7453827
NC	numeric	75.73650592	76.74653773	78	0.462327419
RShankACCX	numeric	0	0	0	0
RShankACCY	numeric	0	0	0	0
RShankACCZ	numeric	0	0	0	0

RShankGYROX	numeric	0	0	0	0
RShankGYROY	numeric	0	0	0	0
RShankGYROZ	numeric	0	0	0	0
NC.1	numeric	0	0	0	0
WaistACCX	numeric	0	0	0	0
WaistACCY	numeric	0	0	0	0
WaistACCZ	numeric	0	0	0	0
WaistGYROX	numeric	0	0	0	0
WaistGYROY	numeric	0	0	0	0
WaistGYROZ	numeric	0	0	0	0
NC.2	numeric	0	0	0	0
ArmACCX	numeric	-12104	-1386.31135	9115.724341	2645.708806
ArmACCY	numeric	-4366	7902.681666	16238.69064	1181.805509
ArmACCZ	numeric	-6670.14448	1095.578985	7863.337376	2250.41035
ArmGYROX	numeric	-6520.97075	60.99911899	8527	500.2248846
ArmGYROY	numeric	-4153	44.25171796	11613.32033	481.9906489
ArmGYROZ	numeric	-3541.15562	11	8299.118246	380.308373
SC	numeric	1865.680132	1917	1963.582499	11.74182746
Label	numeric	0	0	1	0.472175407
Data Summary Report (Patient ID:07)					
column	type	min	median	max	std
TIME	date				
FP1	numeric	-320.0907	-0.27005	256.3568	28.09271105
FP2	numeric	-340.3571	-0.3679	278.1224	30.28969141
F3	numeric	-335.4215	-0.2725	260.7109	28.16222269
F4	numeric	-361.5392	-0.31915	274.49	29.10020584
C3	numeric	-339.3167	-0.2881	270.1392	28.34352955
C4	numeric	-359.4484	-0.38145	282.6016	29.04537456
P3	numeric	-353.2077	-0.4897	268.0963	28.40839343
P4	numeric	-344.1803	-0.33825	265.0492	27.91450941
O1	numeric	-367.5522	-0.8434	275.9131	31.38665991
O2	numeric	-386.6357	-0.0814	272.8847	28.51876367
F7	numeric	-308.0178	-0.2508	250.4309	26.26235459
F8	numeric	-364.8835	-0.3138	288.7246	29.25544697
P7	numeric	-413.6119	-0.019	292.0379	30.69470908
P8	numeric	-368.7953	0.41305	242.2126	28.06867619
Fz	numeric	-338.8179	-0.30945	268.4061	29.13306311
Cz	numeric	-341.528	-0.25045	278.0649	29.08968888
Pz	numeric	-357.1288	-0.51095	281.1572	29.29434684
FC1	numeric	-333.8459	-0.3049	263.9013	28.57550508

FC2	numeric	-352.8013	-0.32455	278.4792	28.97174466
CP1	numeric	-346.0868	-0.3822	277.791	28.35588618
CP2	numeric	-353.2262	-0.36115	279.157	28.5247529
FC5	numeric	-341.306	-0.19455	266.7835	27.50085211
FC6	numeric	-338.5204	0.1027	244.1694	27.27286803
CP5	numeric	-355.242	-0.5111	273.2054	29.32496929
CP6	numeric	-364.9681	-0.50845	274.2427	29.16814034
EMG1	numeric	-5180	65.5	4712.5	1806.002711
EMG2	numeric	-4314.5	59	4011	857.6260378
IO	numeric	-694.5	-1.5	615	82.97356136
EMG3	numeric	-3524	25	3806	446.4090513
EMG4	numeric	-4240	44	4200	1091.292878
LShankACCX	numeric	-10298	8214	29906	2083.094267
LShankACCY	numeric	-39551.7089	3.652358099	20328.72539	4377.84709
LShankACCZ	numeric	-39078.7061	-2527.05634	22919.51621	4424.117948
LShankGYROX	numeric	-10099.7374	92.74937083	8331.096973	1263.291887
LShankGYROY	numeric	-6582.66703	-307.854009	10751.73793	2378.006493
LShankGYROZ	numeric	-7621.47817	205.267067	5177.483708	1942.382781
NC	numeric	75.73875749	77	77.26293399	0.279533152
RShankACCX	numeric	0	0	0	0
RShankACCY	numeric	0	0	0	0
RShankACCZ	numeric	0	0	0	0
RShankGYROX	numeric	0	0	0	0
RShankGYROY	numeric	0	0	0	0
RShankGYROZ	numeric	0	0	0	0
NC.1	numeric	0	0	0	0
WaistACCX	numeric	0	0	0	0
WaistACCY	numeric	0	0	0	0
WaistACCZ	numeric	0	0	0	0
WaistGYROX	numeric	0	0	0	0
WaistGYROY	numeric	0	0	0	0
WaistGYROZ	numeric	0	0	0	0
NC.2	numeric	0	0	0	0
ArmACCX	numeric	-1965.19918	7572	13265.88134	1523.604818
ArmACCY	numeric	-5361	2983	11346.25298	1203.31924
ArmACCZ	numeric	-6723	434.0288888	9685	1718.200817

ArmGYROX	numeric	-8213.79769	72.29643163	4477.895169	824.3664951
ArmGYROY	numeric	-3443.46845	41.07321121	4397	532.0184537
ArmGYROZ	numeric	-6739.65602	8.767330095	4270.432827	558.7778002
SC	numeric	1155.745596	1516.999968	1806	229.2093293
Label	numeric	0	0	1	0.499787293
Data Summary Report (Patient ID:08 OFF1)					
column	type	min	median	max	std
TIME	date				
FP1	numeric	-93.4103	-0.1187	118.4184	13.06653125
FP2	numeric	-85.9918	-0.10565	92.3331	10.68773138
F3	numeric	-81.9989	-0.0664	91.7142	10.27417421
F4	numeric	-80.857	-0.0529	82.1433	9.716013437
C3	numeric	-84.8568	-0.0549	82.8049	10.13770426
C4	numeric	-85.4321	-0.0614	86.8685	10.08146637
P3	numeric	-89.7157	-0.0903	83.0065	11.69854943
P4	numeric	-92.966	-0.05795	85.98	11.24950481
O1	numeric	-106.3742	0.10025	102.6846	15.09512529
O2	numeric	-113.4299	0.0516	106.3769	15.48744194
F7	numeric	-69.2916	0.055	84.6588	8.767311369
F8	numeric	-68.1816	0.08025	92.5752	9.403656421
P7	numeric	-79.8552	0.0893	63.8385	10.18990496
P8	numeric	-98.7337	0.00905	102.1658	11.57132358
Fz	numeric	-83.277	-0.07325	86.7788	10.51179887
Cz	numeric	-74.1189	-0.10985	83.3168	10.24775096
Pz	numeric	-90.6497	-0.0858	84.8525	11.27992676
FC1	numeric	-81.5884	-0.0387	84.8008	10.31718547
FC2	numeric	-262.6229	0.0106	92.3251	12.58948953
CP1	numeric	-81.3926	-0.1156	85.5962	11.04512001
CP2	numeric	-89.8303	-0.0947	86.862	10.86023308
FC5	numeric	-82.0215	0.03015	82.0102	9.997370021
FC6	numeric	-74.695	0.0374	84.3778	8.934887056
CP5	numeric	-80.7551	-0.00475	74.6394	10.02796329
CP6	numeric	-85.3294	-0.03125	85.1838	9.878845131
EMG1	numeric	-4077.5	-12.5	4404	740.6742704
EMG2	numeric	-3670.5	-12	3724	480.3584686
IO	numeric	-482.5	-0.5	590	54.25067813
EMG3	numeric	-3254	35	2866.5	514.314365
EMG4	numeric	-4154	-10.5	4628	734.3102969
LShankACC X	numeric	-22526.9407	8069.115891	36141.89423	2389.104526
LShankACC Y	numeric	-34746.1133	910.6043892	40763.35459	3637.065367
LShankACC Z	numeric	-36986.9422	-2599.06699	31479	1412.866334

LShankGYROX	numeric	-10693.7372	-148.29633	14199.00165	1498.458431
LShankGYROY	numeric	-5328.75128	-17.9691975	6398.049911	402.2356003
LShankGYROZ	numeric	-8887.6392	-68.9411336	11663.7177	2397.652635
NC	numeric	75.73650873	76.03873191	78.09246461	0.431937379
RShankACCX	numeric	0	0	0	0
RShankACCY	numeric	0	0	0	0
RShankACCZ	numeric	0	0	0	0
RShankGYROX	numeric	0	0	0	0
RShankGYROY	numeric	0	0	0	0
RShankGYROZ	numeric	0	0	0	0
NC.1	numeric	0	0	0	0
WaistACCX	numeric	0	0	0	0
WaistACCY	numeric	0	0	0	0
WaistACCZ	numeric	0	0	0	0
WaistGYROX	numeric	0	0	0	0
WaistGYROY	numeric	0	0	0	0
WaistGYROZ	numeric	0	0	0	0
NC.2	numeric	0	0	0	0
ArmACCX	numeric	-16	6846	11745	1555.540021
ArmACCY	numeric	-1612.17966	4727	10475.70191	1028.813486
ArmACCZ	numeric	-2687	-504.208972	8112.767096	1796.138561
ArmGYROX	numeric	-4431.61428	58.31267352	3578	586.7883189
ArmGYROY	numeric	-7858.17777	51.80971321	3874.937259	461.3184298
ArmGYROZ	numeric	-3541.16565	8	5245.966405	486.5107059
SC	numeric	1204.892361	1299.008268	1453.10629	49.60453454
Label	numeric	0	0	1	0.499258785

Data Summary Report (Patient ID:08 OFF2)					
column	type	min	median	max	std
TIME	date				
FP1	numeric	-113.6998	-0.2786	133.142	16.14221896
FP2	numeric	-100.8578	-0.2389	124.6247	14.70023802
F3	numeric	-104.8109	-0.3008	116.7333	15.74530623
F4	numeric	-98.4294	-0.2447	126.5083	14.64746816
C3	numeric	-105.2302	-0.3262	114.4703	16.00025322
C4	numeric	-109.9873	-0.25545	127.1873	15.44462693
P3	numeric	-115.7957	-0.25525	116.4744	16.67788838
P4	numeric	-125.3715	-0.23265	123.4232	16.74357824

O1	numeric	-125.4375	0.062	132.0991	19.6138683
O2	numeric	-131.641	0.05	140.7168	19.52011628
F7	numeric	-102.6127	-0.30095	122.285	15.94509659
F8	numeric	-96.7337	-0.15595	127.1053	14.57656722
P7	numeric	-113.7526	-0.1698	113.3682	15.73450475
P8	numeric	-146.862	-0.08935	134.0083	16.96101125
Fz	numeric	-103.2601	-0.2727	122.5067	15.55849574
Cz	numeric	-114.663	-0.29985	124.0771	16.03425938
Pz	numeric	-116.0114	-0.28545	124.5876	16.38917882
FC1	numeric	-101.3046	-0.2775	118.5294	15.7980095
FC2	numeric	-358.0674	0.0598	194.8398	19.61958276
CP1	numeric	-106.182	-0.32125	115.888	16.15317375
CP2	numeric	-113.2984	-0.2891	126.6162	16.0045926
FC5	numeric	-104.9108	-0.3277	110.7302	16.03181659
FC6	numeric	-103.618	-0.23135	127.0363	14.81531692
CP5	numeric	-108.7995	-0.3252	111.2709	16.36379335
CP6	numeric	-116.6897	-0.28925	126.7342	15.91856339
EMG1	numeric	-3771	-7	3704	489.4972907
EMG2	numeric	-3614.5	-5	3255	268.4163646
IO	numeric	-454	0	416	44.07949082
EMG3	numeric	-2640.5	1	1466.5	399.700362
EMG4	numeric	-3604	-8.5	4019	495.8737177
LShankACCX	numeric	-14904.8118	8152.43369	35025.32596	2216.165915
LShankACCY	numeric	-25930.3361	1435.505639	38493.58774	3545.944654
LShankACCZ	numeric	-22630.5401	-1868	20503	1275.42023
LShankGYROX	numeric	-7705.15728	-139.21409	13322	1360.945905
LShankGYROY	numeric	-4528.37434	5	5685.81057	654.9899345
LShankGYROZ	numeric	-9674.26342	-58.3248826	12349.44213	2430.693885
NC	numeric	75.73686471	77	78.0638303	0.294960791
RShankACCX	numeric	0	0	0	0
RShankACCY	numeric	0	0	0	0
RShankACCZ	numeric	0	0	0	0
RShankGYROX	numeric	0	0	0	0
RShankGYROY	numeric	0	0	0	0
RShankGYROZ	numeric	0	0	0	0
NC.1	numeric	0	0	0	0
WaistACCX	numeric	0	0	0	0

WaistACCY	numeric	0	0	0	0
WaistACCZ	numeric	0	0	0	0
WaistGYROX	numeric	0	0	0	0
WaistGYROY	numeric	0	0	0	0
WaistGYROZ	numeric	0	0	0	0
NC.2	numeric	0	0	0	0
ArmACCX	numeric	-6497.56659	6963.984746	12070.69472	1905.742785
ArmACCY	numeric	-1887	4471.031708	11834.48344	1092.621597
ArmACCZ	numeric	-6390	411.4977601	11407.77541	1597.177875
ArmGYROX	numeric	-7159.20875	65	7977.392124	589.2403032
ArmGYROY	numeric	-5576.21824	47	4838.047639	475.2440198
ArmGYROZ	numeric	-5275.44868	10.15092657	5859.774355	461.032099
SC	numeric	1296.767895	1360	1523.152512	60.05463791
Label	numeric	0	1	1	0.499433017
Data Summary Report (Patient ID:09)					
column	type	min	median	max	std
TIME	date				
FP1	numeric	-86.4327	0.0082	130.4343	12.02300196
FP2	numeric	-91.3354	-0.14815	82.1125	12.09868691
F3	numeric	-92.8167	0.0349	86.8975	11.09412168
F4	numeric	-93.0759	-0.05945	79.8466	11.27399913
C3	numeric	-92.326	0.0703	85.3005	10.37559893
C4	numeric	-92.0222	0.0381	69.899	9.911955194
P3	numeric	-74.4363	-0.0644	111.5722	10.46903319
P4	numeric	-100.7013	-0.0856	69.6058	10.27199897
O1	numeric	-94.1138	-0.18775	88.0249	10.82139181
O2	numeric	-105.7615	-0.11715	86.7596	11.13893792
F7	numeric	-88.2179	0.14815	79.2948	11.40155397
F8	numeric	-100.1	-0.01005	74.3073	10.56380784
P7	numeric	-94.5331	0.0934	72.9107	10.26952669
P8	numeric	-98.9096	-0.00235	61.3393	8.420692966
Fz	numeric	-94.6243	-0.0529	75.4656	10.95213883
Cz	numeric	-95.064	0.0043	73.862	10.33989949
Pz	numeric	-234.6887	-0.0616	429.8066	18.09313245
FC1	numeric	-94.6685	0.0233	81.1596	10.40792677
FC2	numeric	-93.3093	0.01305	93.2507	10.30047435
CP1	numeric	-96.1598	0.00275	79.9403	9.987803003
CP2	numeric	-95.1543	-0.02395	70.0455	10.04324582
FC5	numeric	-91.3014	0.1295	89.3605	10.31459003
FC6	numeric	-98.0398	0.0475	62.6441	9.721379718
CP5	numeric	-91.4326	0.11905	132.1226	9.963334394
CP6	numeric	-95.5412	0.0423	84.2607	9.207885001

EMG1	numeric	-6477.5	-303.5	6501	3224.101051
EMG2	numeric	-5965	-1756	6545.5	3224.283872
IO	numeric	-552.5	-0.5	558	75.68472116
EMG3	numeric	-5636.5	-329	6321.5	3004.816007
EMG4	numeric	-5607	0	5024	1913.570912
LShankACCX	numeric	-9013.9962	8410.712875	33740.25102	2648.659067
LShankACCY	numeric	-38862.1098	-796.888186	26847	3455.366303
LShankACCZ	numeric	-19245.937	-1441.40964	21696	1593.489451
LShankGYROX	numeric	-10655.2177	23.1546604	14745.83737	1522.7577
LShankGYROY	numeric	-4278	-20.0546979	3890.831971	1028.110978
LShankGYROZ	numeric	-12378.6809	282.9111162	7171.969392	3064.812536
NC	numeric	75.73647655	76.99985345	78	0.41195088
RShankACCX	numeric	-4308.33789	8208.342705	34165.1193	2000.084022
RShankACCY	numeric	-45418.1595	886.6361168	38414.01391	3403.672654
RShankACCZ	numeric	-24069.7504	-2112.71062	23646.30583	1571.570711
RShankGYROX	numeric	-12325.0062	-182.212178	16132.73483	1636.276557
RShankGYROY	numeric	-3519.74182	1.422729465	3700.472573	691.7380455
RShankGYROZ	numeric	-7864.58983	-320.928085	11111	2983.484664
NC.1	numeric	50.73875782	52	52.26349623	0.317134531
WaistACCX	numeric	2552.75809	8099.339874	24072.61594	791.3529838
WaistACCY	numeric	-3899.31698	-161.267389	6135.546146	769.3997662
WaistACCZ	numeric	-4283.46704	-736.797949	6082	854.9564691
WaistGYROX	numeric	-3228.86074	22.54579083	3727.21606	907.7406702
WaistGYROY	numeric	-9386.15415	7.652142831	3928.862641	372.254213
WaistGYROZ	numeric	-1231.24361	-13.3028225	1832	263.5448184
NC.2	numeric	2545.907289	2551	2556	1.725392759
ArmACCX	numeric	2139.686814	7931.677285	14139.34447	1124.941507
ArmACCY	numeric	-3823.45639	2051.886809	9025.419867	677.5173434
ArmACCZ	numeric	-15920.8033	20.50244492	10742.23123	1300.680919
ArmGYROX	numeric	-3903.01109	33.75878873	8104.757097	1029.017635
ArmGYROY	numeric	-3451.66058	32.73024072	4271.98484	537.5808405
ArmGYROZ	numeric	-2267.04322	15.32534734	3447.960767	522.9056481
SC	numeric	1138.77384	1500.081295	1690	73.31091703
Label	numeric	0	0	1	0.343107721
Data Summary Report (Patient ID:10)					
column	type	min	median	max	std

TIME	date				
FP1	numeric	-301.1128	0.24325	220.4773	21.69484571
FP2	numeric	-301.276	0.1764	243.2908	22.2908077
F3	numeric	-315.6562	0.07695	221.3869	21.65708468
F4	numeric	-303.2243	0.1129	226.5987	21.38271386
C3	numeric	-311.9753	0.142	221.2077	21.22482363
C4	numeric	-306.1209	0.1337	218.4697	20.67984513
P3	numeric	-307.2547	0.2886	218.7021	20.00232366
P4	numeric	-291.9897	0.45375	211.3981	21.83914013
O1	numeric	-317.6464	0.0934	214.8969	23.34653441
O2	numeric	-395.8026	0.59705	573.0731	36.52035943
F7	numeric	-329.5487	0.0874	223.4014	21.20848831
F8	numeric	-304.0456	0.13705	224.0302	20.27105983
P7	numeric	-330.0252	0.65775	191.0475	21.86523792
P8	numeric	-229.8107	0.4579	230.4947	19.95273388
Fz	numeric	-424.3487	0.11985	220.9714	22.39471363
Cz	numeric	-598.8254	0.12285	220.9998	23.2202985
Pz	numeric	-383.8913	0.389	215.8681	21.24000727
FC1	numeric	-323.6823	0.1467	222.7947	21.31170974
FC2	numeric	-290.833	0.0976	214.797	22.34408274
CP1	numeric	-402.7262	0.1354	213.2583	21.92986558
CP2	numeric	-439.2936	-0.10745	499.7708	34.13844354
FC5	numeric	-311.7401	-0.00515	224.447	21.2755363
FC6	numeric	-296.0965	0.1394	223.7836	20.82398428
CP5	numeric	-310.8072	0.041	225.6814	20.51243671
CP6	numeric	-275.0531	0.15535	214.5261	20.20191027
EMG1	numeric	-6126	-54	6816	2013.828311
EMG2	numeric	-6581	23.5	6006	1767.335508
IO	numeric	-502	0.5	413.5	60.96598688
EMG3	numeric	-5607	7.5	4808	1065.077182
EMG4	numeric	-6076	20	6318.5	2142.636006
LShankACCX	numeric	-14420.1416	8222.271724	37621.0549	2036.643223
LShankACCY	numeric	-37788.0501	-336	30996.86913	2555.499973
LShankACCZ	numeric	-22001	-2233.81657	21778.40269	1189.215412
LShankGYROX	numeric	-8578.11759	-64.3523335	9008.790913	800.500741
LShankGYROY	numeric	-4976.28897	13.93895373	6612.457744	536.125608
LShankGYROZ	numeric	-8919	67.19915652	5901.543303	1609.220487
NC	numeric	75.738744	77	78.19292344	0.185766103
RShankACCX	numeric	-7793.31768	8107.009027	37310.11541	2299.734412

RShankACCY	numeric	-42827.2108	1979.168354	37097.00373	3224.352299
RShankACCZ	numeric	-26599	-1524	24790	1680.440012
RShankGYROX	numeric	-11657.3543	-25.2747268	10443	944.0701754
RShankGYROY	numeric	-6215.31948	27.47625606	5582.423974	1017.14178
RShankGYROZ	numeric	-6346.95894	-71.9748101	10158.15441	1782.644703
NC.1	numeric	50.74933904	52	52.25515569	0.135147812
WaistACCX	numeric	5032.531257	8050.940129	12511.18318	421.5780068
WaistACCY	numeric	-3264	1413.414933	5935.010883	661.1854692
WaistACCZ	numeric	-3805.64539	-852.326446	2980.702624	627.6955249
WaistGYROX	numeric	-2773.68869	17.8186745	2488.482886	432.1245937
WaistGYROY	numeric	-2774.04694	17.39724009	2031.967329	296.5567483
WaistGYROZ	numeric	-1300.91925	-14.9427535	1183.442555	179.6975707
NC.2	numeric	2536.8132	2563.945661	2573	12.00585744
ArmACCX	numeric	-9976.03228	7976	31993.38665	1671.181942
ArmACCY	numeric	-7738	374.3377112	32724.88281	1946.736207
ArmACCZ	numeric	-12417.7487	513.185304	17768.47985	1894.067786
ArmGYROX	numeric	-9753.46056	70.92185255	9804.934568	927.0298461
ArmGYROY	numeric	-7358.13641	32.19114381	7344.213294	547.1198893
ArmGYROZ	numeric	-10799.0183	27.06492786	9526.548989	684.9620621
SC	numeric	1310	1474.638566	1822	64.3716367
Label	numeric	0	0	1	0.499455051
Data Summary Report (Patient ID:11)					
column	type	min	median	max	std
TIME	date				
FP1	numeric	-132.9354	0.8484	135.6611	19.93982393
FP2	numeric	-137.1573	0.674	143.0034	21.39614439
F3	numeric	-137.4919	0.89415	139.7266	20.42768385
F4	numeric	-127.0333	0.7177	135.917	19.67884275
C3	numeric	-140.758	0.9029	131.0614	20.54204278
C4	numeric	-126.895	0.488	122.4739	18.78177352
P3	numeric	-151.8693	0.831	136.0338	21.21734322
P4	numeric	-138.0259	0.70835	134.0694	20.73466765
O1	numeric	-233.2174	0.6158	223.2135	32.33438806
O2	numeric	-234.9211	-0.1869	227.0902	37.06216776
F7	numeric	-136.587	1.03655	137.5405	19.86445876
F8	numeric	-118.0236	0.3681	116.5399	17.14232707
P7	numeric	-172.4168	0.787	142.2368	21.34980867
P8	numeric	-147.0612	-0.24215	118.1641	17.48748686
Fz	numeric	-135.0135	0.72065	140.532	21.21387752

Cz	numeric	-133.2341	0.6822	131.809	20.83761298
Pz	numeric	-143.561	0.7327	130.0948	21.91051614
FC1	numeric	-139.7289	0.81825	133.4445	21.13293471
FC2	numeric	-142.9053	0.5535	135.9266	20.89374383
CP1	numeric	-144.0014	0.73885	131.3062	20.981315
CP2	numeric	-177.7331	0.6095	128.0977	23.8026927
FC5	numeric	-136.5191	0.92365	135.9039	19.80600875
FC6	numeric	-124.9219	0.2796	113.3084	17.48789228
CP5	numeric	-149.82	0.86065	140.3595	19.98385789
CP6	numeric	-153.3648	0.13155	116.2557	18.0830916
EMG1	numeric	-7162.5	1042	6570	3075.121069
EMG2	numeric	-7382	579	7270.5	2897.246981
IO	numeric	-626.5	-1	750.5	69.87825224
EMG3	numeric	-5171.5	108.5	4586.5	2042.069014
EMG4	numeric	-7429.5	324	7107.5	2793.023743
LShankACCX	numeric	-5201.28629	8072.38494	33434.88625	1377.042587
LShankACCY	numeric	-33004.138	-2246.48957	19283.55973	1768.523583
LShankACCZ	numeric	-22110	-2224.70561	15948.84458	1018.412096
LShankGYROX	numeric	-9488.57795	118	7785	662.7528126
LShankGYROY	numeric	-3531.54613	-25.6111466	4387.227824	595.46732
LShankGYROZ	numeric	-8245.73775	153.3091588	8750.574628	1687.722869
NC	numeric	2592.81316	2601	2619.615341	4.947588847
RShankACCX	numeric	-16817.7675	8062.538559	35410.4828	1319.242171
RShankACCY	numeric	-42444.3689	2167.650847	37769.02993	2155.859266
RShankACCZ	numeric	-24747.3015	-2218.56817	14753.90992	1007.293852
RShankGYROX	numeric	-6558.42053	-46	10786.16264	889.3921587
RShankGYROY	numeric	-5223.34944	60.7199769	3828.491441	477.2121923
RShankGYROZ	numeric	-6485.81935	-155	9448.805567	1864.757766
NC.1	numeric	2599.741345	2603.999391	2617.221955	4.051098518
WaistACCX	numeric	0	0	0	0
WaistACCY	numeric	0	0	0	0
WaistACCZ	numeric	0	0	0	0
WaistGYROX	numeric	0	0	0	0
WaistGYROY	numeric	0	0	0	0
WaistGYROZ	numeric	0	0	0	0
NC.2	numeric	0	0	0	0

ArmACCX	numeric	-471.775417	7647.112115	13278.60908	1485.74114
ArmACCY	numeric	-3621.31192	2597.985233	15227.43104	2083.686584
ArmACCZ	numeric	-8803.94127	2338.901636	11179.00973	2901.268324
ArmGYROX	numeric	-4427	10	5888.22955	916.3054638
ArmGYROY	numeric	-6604	33.85201697	5182	509.0040876
ArmGYROZ	numeric	-3380.23775	40.34243482	3908.589946	426.1800719
SC	numeric	1733.88528	1795.041638	1862.229482	22.24221466
Label	numeric	0	0	1	0.491351292
Data Summary Report (Patient ID:12)					
column	type	min	median	max	std
TIME	date				
FP1	numeric	-169.2328	0.14445	129.2307	21.58627586
FP2	numeric	-150.4173	0.11325	141.4324	20.40904324
F3	numeric	-161.624	0.1689	131.6173	21.82899022
F4	numeric	-159.323	0.05905	131.1526	20.82708922
C3	numeric	-168.4767	0.24495	127.4585	21.72532214
C4	numeric	-149.7637	0.14475	131.282	19.98337664
P3	numeric	-168.1267	0.2701	134.632	22.23840357
P4	numeric	-151.1962	0.17925	140.7234	20.30442071
O1	numeric	-188.2674	0.18605	160.9038	22.59323641
O2	numeric	-172.1627	0.0436	159.8456	22.91897129
F7	numeric	-166.6165	0.18815	133.4966	21.82297351
F8	numeric	-140.4719	0.05505	122.0282	19.01222564
P7	numeric	-170.9383	0.29355	137.4575	21.87334435
P8	numeric	-182.1227	0.01395	121.5078	17.45928545
Fz	numeric	-165.2733	0.14105	133.4353	21.67178258
Cz	numeric	-163.206	0.1766	132.974	21.88970022
Pz	numeric	-164.337	0.23195	127.5475	21.58211925
FC1	numeric	-163.2094	0.2026	128.3819	21.93777812
FC2	numeric	-162.4922	0.123	135.3177	21.18363874
CP1	numeric	-166.306	0.2146	125.6577	21.72560729
CP2	numeric	-156.313	0.1936	130.1373	21.11025188
FC5	numeric	-164.6552	0.2128	129.1928	21.6876705
FC6	numeric	-162.1382	0.0345	120.9305	19.09097353
CP5	numeric	-169.1654	0.25625	133.8133	21.85755663
CP6	numeric	-169.8292	0.0798	113.6245	18.04755725
EMG1	numeric	-7170.5	-554	7376.5	2713.130687
EMG2	numeric	-5812.5	-59	5980.5	2628.47139
IO	numeric	-336.5	0	369	40.96162702
EMG3	numeric	-3981	-64.5	4279	1070.914397
EMG4	numeric	-6578.5	22	6507	2787.083107
LShankACCX	numeric	0	0	0	0

LShankACCY	numeric	0	0	0	0
LShankACCZ	numeric	0	0	0	0
LShankGYROX	numeric	0	0	0	0
LShankGYROY	numeric	0	0	0	0
LShankGYROZ	numeric	0	0	0	0
NC	numeric	0	0	0	0
RShankACCX	numeric	-3242	8016.41028	28591.25205	1136.349505
RShankACCY	numeric	-34369.341	2166.075389	34457.42048	2750.724532
RShankACCZ	numeric	-22947.0355	-2573.13947	12072.52156	1035.654073
RShankGYROX	numeric	-7467.59744	-56.7732796	10051.39156	887.9966953
RShankGYROY	numeric	-2840.70346	40.03984963	2929	432.8196436
RShankGYROZ	numeric	-5351.1438	-179.404334	9947.539836	1727.57936
NC.1	numeric	2625.738718	2631.120976	2639	4.408992119
WaistACCX	numeric	4533.671538	8512.646764	14818.28259	655.6301494
WaistACCY	numeric	-1888.50348	1828.178227	6084	662.5011923
WaistACCZ	numeric	-5477.69647	2275.574234	7619.65416	1565.889256
WaistGYROX	numeric	-2414.02127	-43.4230614	2237.979246	421.1999613
WaistGYROY	numeric	-3836	-26	2411	242.4235823
WaistGYROZ	numeric	-1124.33168	61.22595576	1112.873261	205.8430793
NC.2	numeric	2626.738761	2630	2637.260856	2.207300438
ArmACCX	numeric	-4152.24474	8199.62529	13016.0713	2936.037043
ArmACCY	numeric	-6658	2642.679078	12285.37308	1759.452625
ArmACCZ	numeric	-12060	-534.576728	11509.25783	2524.232281
ArmGYROX	numeric	-9006	-16.7969817	6372.908957	659.3987618
ArmGYROY	numeric	-4125	15.77916202	4701.014713	410.302446
ArmGYROZ	numeric	-5258	29.06558809	3950.297126	546.2422919
SC	numeric	1752.62459	1945	1966.143454	9.802259707
Label	numeric	0	0	1	0.425237579

Noise Proportion & SNR

In this part, we calculate Noise Proportion and the SNR(Signal to Noise Ratio). SNR is defined as the logarithm of the ratio of the noise-reduced signal power to the noise signal power[1]. And the noise proportion is defined as the ratio of the noise signal points and the original signal points. The artifact removal is done through EEGLAB.

Noise Proportion				
Patient ID	Task1	Task2	Task3	Task4
Patient ID:01	0.030309859	0.016503067	0.05826087	0.3232
Patient ID:02	0.009873418	0.017253219	0.421	0.440869565
Patient ID:03	0.006276347	0.01392	0.459142857	0.233623188
Patient ID:04	0.075714286	0.026553672	0.368571429	0.154615385
Patient ID:05	0.097478992	0.537333333	0.21	0.753846154
Patient ID:06	0.102857143	0.171130064	0.016666667	0.202333333
Patient ID:07	0.172783505	0.179928826	0.615	0.5
Patient ID:08 OFF1	0.046774942	0.057728532	0.175909091	0.199090909
Patient ID:08 OFF2	0.211444142	0.21902439	0.316363636	0.416
Patient ID:09	0.075416667	0.082672414	0.041449275	0.336470588
Patient ID:10	0.101779449	0.152978056	0.447837838	0.240273973
Patient ID:11	0	0	0.033666667	0
Patient ID:12	0	0.041944444	0.07147541	0

	SNR			
Patient ID	Task1	Task2	Task3	Task4
Patient ID:01	110.8885455	110.6618933	78.63279705	83.16044159
Patient ID:02	99.25573387	98.08038898	66.1069982	69.43019962
Patient ID:03	111.8560741	95.28787075	73.01825369	75.53791765
Patient ID:04	76.23312899	82.19018126	63.07794013	65.46151587
Patient ID:05	85.60684996	63.14105246	64.11005239	61.23392943
Patient ID:06	105.6223253	109.7572554	85.07720516	86.01338238
Patient ID:07	98.64132179	86.09096525	79.33516994	82.41795508
Patient ID:08 OFF1	115.477036	112.7573476	87.09529335	76.3692205
Patient ID:08 OFF2	123.8901578	127.2971879	105.5745685	97.80457325
Patient ID:09	86.5463015	102.9726807	108.2469775	74.05038223
Patient ID:10	130.5670294	133.2812201	99.90970309	103.998618

Patient ID:11	102.5087112	99.44681768	76.86200535	79.67180157
Patient ID:12	105.1791185	92.34349968	85.93653877	101.8398265

EEG Quality Index

In this part, we calculate EEG Quality Index including six metrics that are shown to be highly sensitive to various typical artifacts. The first 100 seconds data which were divided into 100 windows from each patient were used for testing. Column 'EQI - Mean Power 1-50Hz' shows the average single-sided amplitude spectrum (1-50Hz range). Column 'EQI - Mean Power 49-51Hz' shows the average single-sided amplitude spectrum (49-51Hz range). Column 'EQI - RMS Amplitude' shows the root-mean-square (RMS) amplitude of the EEG signal. Column 'EQI - Max Gradient' shows the largest difference between all adjacent samples within the window. Column 'EQI - Zero-crossing Rate' shows the rate at which the signal changes signs from positive to negative. Column 'EQI - Kurtosis' shows the Kurtosis of the data. Kurtosis is a standard statistical measure of the heaviness of the tails of the distribution of samples. Each metric contributes equally to the EQI, with higher scores relating to lower-quality signals [2].

Patients ID	EQI - Mean Power 1-50Hz	EQI - Mean Power 49-51Hz	EQI - RMS Amplitude	EQI - Max Gradient	EQI - Zero-crossing Rate	EQI - Kurtosis
Patient ID:01	0.271554918	0.325915032	0.281182198	0.301285227	0.509187289	0.210763419
Patient ID:02	0.459656977	0.362971253	0.417530959	0.362891255	0.490472516	0.281742995
Patient ID:03	0.370059595	0.343500939	0.364819297	0.36283065	0.504658088	0.274393649
Patient ID:04	0.363624169	0.327182077	0.229389305	0.355114442	0.478939819	0.306046342
Patient ID:05	0.413590837	0.346903699	0.390897154	0.339143048	0.56370336	0.319002848
Patient ID:06	0.362598735	0.324631018	0.355970182	0.352557726	0.501484818	0.305895234
Patient ID:07	0.291922991	0.299427486	0.227986505	0.24594493	0.486519464	0.280937355
Patient ID:08 OFF1	0.344175673	0.326202703	0.242146219	0.295621503	0.482770853	0.283502151
Patient ID:08 OFF2	0.284423345	0.285632209	0.245131209	0.183272257	0.498330943	0.179187491
Patient ID:09	0.257185107	0.342478738	0.272851862	0.254714046	0.509860407	0.28663825
Patient ID:10	0.473510434	0.351685016	0.311761783	0.390901194	0.502174097	0.313177512
Patient ID:11	0.372575908	0.30714127	0.377069554	0.358907497	0.51709824	0.328714167
Patient ID:12	0.281071494	0.332950849	0.257786307	0.336896628	0.494400517	0.226654815

References

[1] Ball, Tonio, et al. "Signal quality of simultaneously recorded invasive and non-invasive EEG." Neuroimage 46.3 (2009): 708-716.
[2] Fickling, Shaun D., et al. "Good data? The EEG quality index for automated assessment of signal quality." 2019 IEEE 10th Annual Information Technology, Electronics and Mobile Communication Conference (IEMCON). IEEE, 2019.
