# Supplementary material for: Multimodal Data for the Detection of Freezing of Gait in Parkinson’s Disease
Source: Sci Data. 2022 Oct 7;9:606. doi: 10.1038/s41597-022-01713-8 (PMC9546845; doi:10.1038/s41597-022-01713-8)
Supplement: Supplementary file 5 — Supplemental Table 1 [file 41597_2022_1713_MOESM5_ESM.pdf]

Supplemental Table 1. Duration of FOG events.

	Duration (s)										
Patient ID	1-5	6-10	11-15	16-20	21-25	26-30	31-35	36-40	41-45	46-50	50+
01	6	5	7	1	1	0	2	0	0	0	0
02*	1	0	0	0	0	0	0	0	0	0	0
03	16	10	6	3	2	0	2	3	0	0	0
04	5	7	2	0	1	0	0	0	0	0	0
05*	0	0	0	0	0	0	0	0	0	0	0
06	1	4	5	7	3	1	1	0	0	0	0
07	10	6	10	5	1	1	0	0	0	0	0
08(1)	11	3	2	1	2	2	1	0	0	2	1
08(2)	4	6	5	3	0	1	2	1	0	2	0
09	38	1	0	0	0	0	0	0	0	0	0
10	4	9	8	1	2	2	0	0	4	1	4
11	25	16	7	2	1	1	2	0	1	0	0
12	2	2	1	2	1	1	1	0	1	1	0
Total	123	69	53	25	14	9	11	4	6	6	14

A star next to the patient ID indicates that there almost no FOG appeared during the experiment. Patient 08 repeated the data collection twice
