# Supplementary material for: Multimodal Data for the Detection of Freezing of Gait in Parkinson’s Disease
Source: Sci Data. 2022 Oct 7;9:606. doi: 10.1038/s41597-022-01713-8 (PMC9546845; doi:10.1038/s41597-022-01713-8)
Supplement: Supplementary file 6 — Supplemental Table 3 [file 41597_2022_1713_MOESM6_ESM.pdf]

Some acceleration data of patients are not available because of device failure during the experiments. The details are shown in the following table. All other data (EEG, EMG, SC) of this experiment are complete.

Supplemental Table 3. Acceleration Data Completeness

Patient ID	LShankACC	RShankACC	WaistACC	ArmACC
1	A	NA	A	A
2	NA	A	A	NA
3	A	NA	A	A
4	NA	A	NA	A
5	A	NA	NA	A
6	A	NA	NA	A
7	A	NA	NA	A
008-1	A	NA	NA	A
008-2	A	NA	NA	A
9	A	A	A	A
10	A	A	A	A
11	A	A	NA	A
12	NA	A	A	A

* LShankACC => Acceleration data on the left shank
RShankACC => Acceleration data on the right shank
WaistACC =>Acceleration data on the waist
ArmACC =>Acceleration data on the arm
A=>Data is Available
NA=>Data is Not Available
